# CB1 Receptor-Dependent and Independent Induction of Lipolysis in Primary Rat Adipocytes by the Inverse Agonist Rimonabant (SR141716A)

**DOI:** 10.3390/molecules25040896

**Published:** 2020-02-18

**Authors:** Günter A. Müller, Andreas W. Herling, Susanne Wied, Timo D. Müller

**Affiliations:** 1 Institute for Diabetes and Obesity (IDO), Helmholtz Diabetes Center (HDC), Helmholtz Zentrum München, German Research Center for Environmental Health (GmbH), 85764 Oberschleissheim, Germany; timo.mueller@helmholtz-muenchen.de; 2 German Center for Diabetes Research (DZD), 85764 Oberschleissheim, Germany; 3 Ludwig-Maximilians-University Munich, Department Biology I, Genetics, 82152 Planegg-Martinsried, Germany; 4 Sanofi Pharma Germany GmbH, Diabetes Research, 65926 Frankfurt am Main, Germany; andreas.herling@sanofi.com (A.W.H.); susanne.wied@sanofi.com (S.W.); 5 Department of Pharmacology and Experimental Therapy, Institute of Experimental and Clinical Pharmacology and Toxicology, Eberhard Karls University Hospitals and Clinics, 72074 Tübingen, Germany

**Keywords:** cyclic adenosine monophosphate (cAMP)-dependent signaling, cannabinoid receptor 1 (CB1R), hormone-sensitive lipase (HSL), interfacial activation, lipid droplets (LD), lipolysis, obesity, Rimonabant

## Abstract

(1) Background: Acute administration of the cannabinoid receptor 1 (CB1R) inverse agonist Rimonabant (SR141716A) to fed Wistar rats was shown to elicit a rapid and short-lasting elevation of serum free fatty acids. (2) Methods: The effect of Rimonabant on lipolysis in isolated primary rat adipocytes was studied to raise the possibility for direct mechanisms not involving the (hypothalamic) CB1R. (3) Results: Incubation of these cells with Rimonabant-stimulated lipolysis to up to 25% of the maximal isoproterenol effect, which was based on both CB1R-dependent and independent mechanisms. The CB1R-dependent one was already effective at Rimonabant concentrations of less than 1 µM and after short-term incubation, partially additive to β-adrenergic agonists and blocked by insulin and, in part, by adenosine deaminase, but not by propranolol. It was accompanied by protein kinase A (PKA)-mediated association of hormone-sensitive lipase (HSL) with lipid droplets (LD) and dissociation of perilipin-1 from LD. The CB1R-independent stimulation of lipolysis was observed only at Rimonabant concentrations above 1 µM and after long-term incubation and was not affected by insulin. It was recapitulated by a cell-free system reconstituted with rat adipocyte LD and HSL. Rimonabant-induced cell-free lipolysis was not affected by PKA-mediated phosphorylation of LD and HSL, but abrogated by phospholipase digestion or emulsification of the LD. Furthermore, LD isolated from adipocytes and then treated with Rimonabant (>1 µM) were more efficient substrates for exogenously added HSL compared to control LD. The CB1R-independent lipolysis was also demonstrated in primary adipocytes from fed rats which had been treated with a single dose of Rimonabant (30 mg/kg). (4) Conclusions: These data argue for interaction of Rimonabant (at high concentrations) with both the LD surface and the CB1R of primary rat adipocytes, each leading to increased access of HSL to LD in phosphorylation-independent and dependent fashion, respectively. Both mechanisms may lead to direct and acute stimulation of lipolysis at peripheral tissues upon Rimonabant administration and represent targets for future obesity therapy which do not encompass the hypothalamic CB1R.

## 1. Introduction

Lipid messengers of the endocannabinoid class play a crucial role in body weight control through engagement of several centrally and peripherally operating molecular mechanisms that coordinate the maintenance of energy homeostasis (for a review see [1]). Initially, endocannabinoids were thought to act exclusively or at least predominantly through the brain via binding to the G-protein-coupled cannabinoid type 1 and 2 receptors (CB1/2R) in the hypothalamus [2]. However, CB1/2R have meanwhile been recognized to be expressed in virtually all body tissues, among them adipocytes of white adipose tissue [3,4]. Importantly, hyperactivation of CB1R downstream signaling correlates with obesity and type 2 diabetes, whereas its blockade by the first-generation CB1R inverse agonist Rimonabant, also named SR141716A by the original manufacturer, leads to considerable lowering of the body weight in laboratory animals and obese patients [5]. Unfortunately, the therapeutic benefit of Rimonabant is severely limited by unwanted neuropsychiatric side effects [6]. As a consequence, cannabinoid research of the past two decades has also considered the involvement of additional or alternative targets and mechanisms operating at peripheral tissues rather than the brain and mediating or contributing to the endocannabinoid-controlled regulation of the feeding behavior and whole body energy metabolism.

One of the first studies arguing for body weight control by endocannabinoids through occupancy by peripheral CB1R demonstrated the lean phenotype, resistance towards diet-induced obesity and unimpaired insulin sensitivity after high fat-feeding of CB1R knockout mice, arguing for functional deficits in the pancreas, liver and adipose tissue [7]. Concomitantly, multiple effects of CB1R agonists upon incubation with primary or cultured cells of peripheral organs, such as stimulation of lipogenesis in adipocytes [7], have been reported. Moreover, prominent expression of the endocannabinoid-degrading enzymes in adipose tissue and liver [8,9] as well as considerable levels of endocannabinoids in these organs, related to the nutritional state [10,11,12,13], have been measured. Strikingly, several investigations with mice and rats strongly suggested that part of the long-lasting body weight-decreasing and acute blood glucose-, insulin- and leptin-lowering activities of Rimonabant upon chronic administration is not mediated by central downregulation of food intake [14,15,16]. As the major conclusion from these results, one current line of obesity research focuses on the design of inverse agonists and antagonists which address the CB1R in peripheral tissues with sufficient selectivity towards the brain. This treatment regimen for obesity would circumvent the danger of central psychotropic side effects in response to blocked endocannabinoid signaling in the hypothalamus [17].

The previous findings that (i) lowering of adipose mass by Rimonabant in diet-induced obese mice is due to upregulated adipose tissue lipolysis leading to increased fatty acid oxidation and TCA cycling, elevated energy expenditure through futile cycling and improved glucose homeostasis [18] and that (ii) enhanced whole-body energy expenditure and lowering of plasma lipid levels is correlated to elevated brown adipose tissue thermogenesis and decreased LD size [19] strongly argued for an additional mode of pharmacological action of Rimonabant at peripheral rather than central sites. However, they did not deal with the question as to whether the control of lipolysis would be initiated by (i) central or peripheral mechanisms and by (ii) CB1R-dependent or independent mechanisms. Furthermore, previous studies have not clarified whether the effects of Rimonabant on adipose gene expression solely depend on centrally initiated processes or the direct interaction of Rimonabant with regulatory mechanisms at adipocytes, or a concerted action between central and adipocyte sites.

Previously, the effect of Rimonabant on the fat distribution was studied in female candy-fed Wistar rats [20]. Candy-diet-induced increases in body weight, total fat mass and visceral as well as skeletal intramyocellular fat were completely reversed in Rimonabant-treated compared to pair-fed rats. Strikingly, these effects, which were maintained during the complete treatment period, were accompanied by only moderate and transient declines in food intake. Thus, other mechanisms, most likely upregulation of lipolysis and fatty acid oxidation according to the available dataset, have been suggested to mediate the improvement of the lipid parameters [20]. Subsequently, this conclusion was corroborated by the finding that Rimonabant causes a dose-dependent, yet moderate increase in basal lipolysis in fed Wistar rats after acute administration (Herling A.W., Müller G.A., Schmoll D.; unpublished data). In the present study, the possibility of a direct effect of Rimonabant in adipose cells, i.e., initiated and mediated by adipocyte instead of brain target sites and mechanisms, with the consequence of lipolysis control was investigated. In fact, Rimonabant in the µM-range significantly stimulated lipolysis in isolated rat adipocytes by engagement of two distinct molecular mechanisms, one dependent on, the other independent of CB1R. They may be regarded as potential targets for future anti-obesity drugs which do not operate *via* central processes.

## 2. Results

### 2.1. Rimonabant Stimulates Lipolysis in Primary Rat Adipocytes

**Figure 1:**
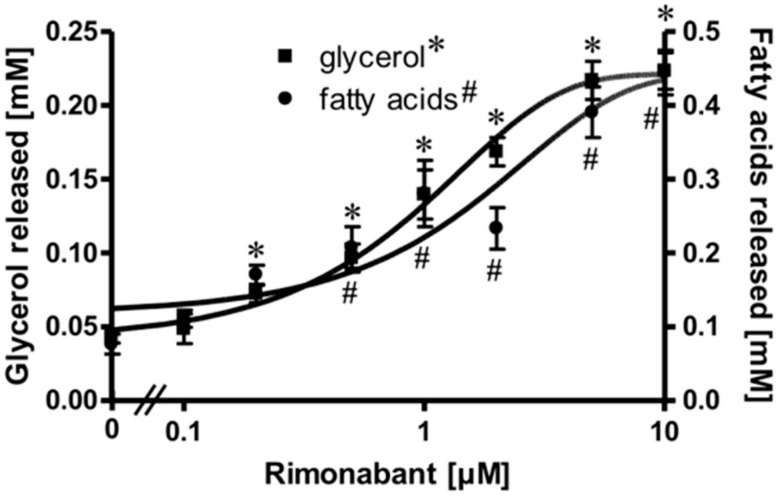
Stimulation of adipocyte lipolysis by Rimonabant. Primary rat adipocytes were incubated (3 h, 37 °C) in the absence or presence of increasing concentrations of Rimonabant. The concentrations of glycerol (■) and (fatty acids) FA (●) released into the incubation medium were assayed enzymatically. Mean ± SD of three different cell preparations with incubations in triplicate and measurements in duplicate. *,^#^ *p* ≤ 0.01 vs. absence of Rimonabant.

Isolated rat adipocytes in primary culture are known to exhibit an exquisite sensitivity and responsiveness to lipolysis regulation by the ß-adrenergic, adenosine and insulin receptors and the corresponding ligands [21,22,23]. These cells were used to study a putative direct effect of the inverse CB1R agonist Rimonabant on peripheral fat tissue lipolysis. For this, the adipocytes were treated with Rimonabant under various conditions and then analyzed for the release of glycerol/fatty acids (FA) as well as for the engagement of known molecular mechanisms regulating lipolysis. Upon treatment of the adipocytes with Rimonabant for 2 h, the concentrations of glycerol and FA in the incubation medium were significantly increased in a concentration-dependent fashion by up to 5-fold with EC_50_ of 0.95 (glycerol) and1.33 (FA) µM (Figure 1). The FA/glycerol ratio of around two at each concentration indicated considerable re-esterification operating in adipocytes under these conditions, the degree to which is apparently not affected by Rimonabant.

**Figure 2:**
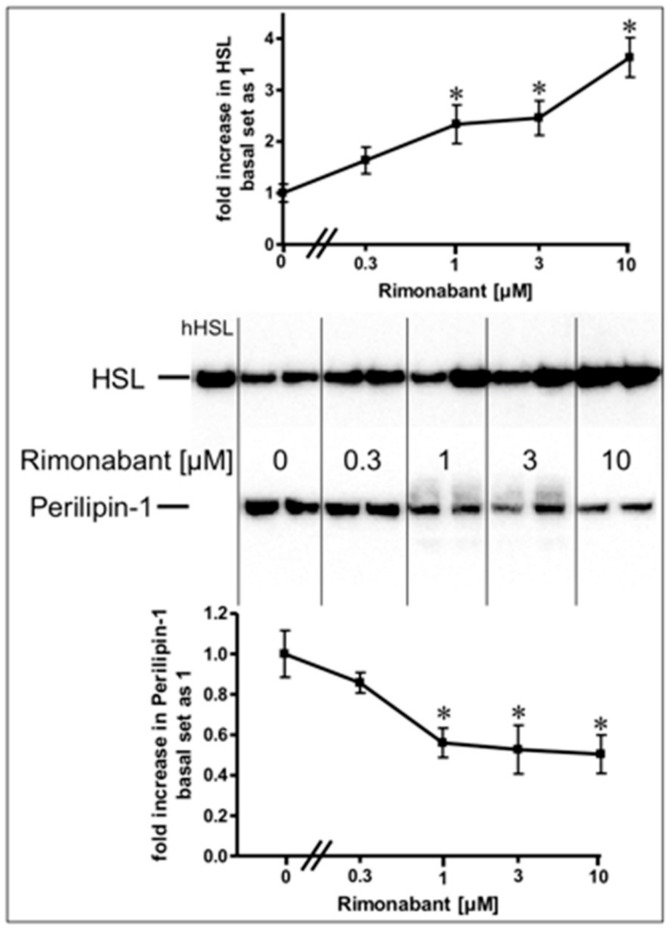
Stimulation of the translocation of hormone-sensitive lipase (HSL) and perilipin-1 by Rimonabant during adipocyte lipolysis. Primary rat adipocytes were incubated (20 min, 37 °C) in the absence or presence of increasing concentrations of Rimonabant. Subsequently, LD were prepared from the homogenate of the washed adipocytes by centrifugation through a sucrose cushion (see Materials and Methods). The top fractions were extracted for removal of lipids. Proteins were then separated by sodium dodecylsulfate polyacrylamide gel electrophoresis (SDS-PAGE) and analyzed for HSL and perilipin-1 by immunoblotting. The images of a typical experiment with electrophoretic runs in duplicate and repeated two times are shown with similar results. hHSL, recombinant human HSL was run in parallel as marker. The quantitative evaluation of the three experiments is shown (mean ± SD). * *p* ≤ 0.05 vs. absence of Rimonabant.

Stimulation of lipolysis in rodent adipocytes by physiological modulators (e.g., catecholamines) is known to rely on the translocation of the hormone-sensitive lipase (HSL) from the cytoplasm to the surface of lipid droplets (LD). This is accompanied by a structural rearrangement of the LD surface protein, perilipin-1, and/or its reverse translocation from the LD to the cytoplasm [24,25,26,27]. The association of HSL and perilipin-1 with LD was assessed with LD prepared from the incubated adipocytes. Incubation with Rimonabant for 20 min significantly increased and decreased the amounts of LD-associated HSL and perilipin-1, respectively, in a concentration-dependent fashion with EC_50_ of 2.46 µM and IC_50_ of 0.72 µM, respectively, reaching a maximal effect at 10 µM and above (Figure 2, results obtained with concentrations above 10 µM not shown). The cellular distribution of other major LD-associated proteins and lipids was not affected by Rimonabant (Supplementary Figure S1), arguing for the specificity of the Rimonabant-induced translocation of HSL and perilipin-1 to/from LD. Moreover, immunoblot analysis of total membrane and cytoplasmic fractions derived from the adipocyte homogenate upon centrifugation through a sucrose cushion (for removal of the floating LD) as pellet and supernatant (recovered below the sucrose cushion) fractions, respectively, for typical subcellular marker proteins did not reveal significant changes in the amounts of CD73, Gce1, caveolin-1 and GLUT1 (cell surface and plasma membrane proteins) as well as of glycerinaldehyde-3-phosphate dehydrogenase (GAPDH) and vimentin (cytoplasmic proteins) in response to treatment (20 min) of primary rat adipocytes with Rimonabant (at 1 and 10 µM) compared to control (data not shown). This finding demonstrated the specificity of the Rimonabant-induced protein redistribution in adipocytes since as far as studied typical LD-associated polypeptides become translocated, only.

### 2.2. Rimonabant Stimulates Lipolysis by CB1R-Dependent and Independent Mechanisms

**Figure 3:**
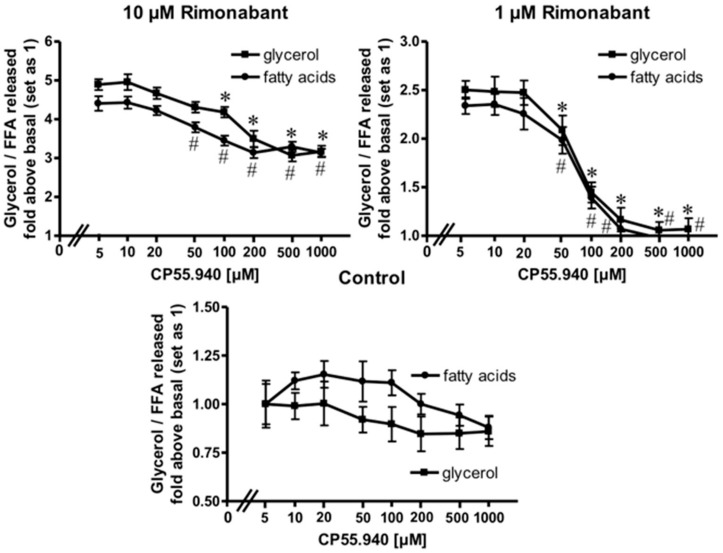
Blockade of the Rimonabant-induced adipocyte lipolysis by a CB1R agonist. Primary rat adipocytes were incubated (20 min, 37 °C) in the absence or presence of increasing concentrations of the CB1R agonist, CP55.940, prior to the addition of Rimonabant (final concentration 10 or 1 µM) or dimethyl sulphoxide (DMSO) (Control). After further incubation (3 h, 37 °C), the concentrations of glycerol (■) and fatty acids (●) released into the incubation medium were assayed enzymatically and calculated as fold increases above basal (absence of Rimonabant set as 1). Mean ± SD of incubations in triplicate and measurements in duplicate. * (glycerol),^#^ (fatty acids) *p* ≤ 0.05 vs. absence of CP55.940; no significant differences with control adipocytes (absence of Rimonabant).

Expression of CB1R has been demonstrated previously in adipose tissue from lean and, more pronouncedly, in obese Zucker (fa/fa) rats [28] as well as in human primary mature adipocytes and subcutaneous adipose tissue from normal and obese women [29]. To assess whether the apparent lipolytic activity of Rimonabant in primary rat adipocytes is mediated by the CB1R, the effect of the potent CB1R agonist, CP55.940, on Rimonabant-induced lipolysis was investigated. The release of glycerol and FA induced by Rimonabant was significantly reduced by excess of CP55.940 in concentration-dependent fashion (Figure 3). Tenfold molar excess of CP55.940 relative to Rimonabant caused suppression of the maximal Rimonabant-induced (10 µM) lipolysis by 50% and half-maximal Rimonabant-induced (at 1 µM) lipolysis was completely blocked by a 200-fold molar excess of CP55.490 (Figure 3). The competition data suggest that up to a concentration of 1 µM, the Rimonabant-induced lipolysis is mediated predominantly by specific interaction with the CB1R. At concentrations above 1 µM roughly half of the lipolytic response seems to be based on CB1R-independent mechanism(s). The CB1R agonist had no significant effect on basal lipolysis (Figure 3, Control), compatible with efficient suppression of lipolysis during isolation of the adipocytes. Moreover, the differentiation of a rapid-onset and slow-onset lipolytic activity of Rimonabant, which is competed for or not, respectively, by CP55.490 is also explained best by the involvement of CB1R-dependent as well as independent mechanisms (Supplementary Figure S2).

Interestingly, the use of two other CB1R antagonists, AM281 and AM251, which act as inverse agonists *in vitro* and display close structural similarity to Rimonabant, led to almost identical results with regard to lipolysis induction by 1 and 10 µM and its complete and partial inhibition, respectively, by CP55.940 (data not shown). In contrast, the cannabinoid receptor 2 inverse agonist, AM630, characterized by more than 150-fold selectivity over CB1R, did not significantly stimulate lipolysis at 0.1–0.5 µM. At 1 and 10 µM, AM630 stimulated lipolysis to about 25 and 90%, respectively, of the Rimonabant effect with 65 and 15% reduction, only (compared to 100 and 50% for Rimonabant) by simultaneous presence of 200 µM of CP55.940. These findings suggest that the structural features required for lipolysis induction (in CB1R-dependent fashion) at concentrations below 1 µM are more stringent and specific than those operating at higher concentrations (in CB1R-independent fashion). Thus the CB1R-independent lipolysis induction by CB1R inverse agonists does apparently not rely on the authentic structure of Rimonabant.

**Figure 4:**
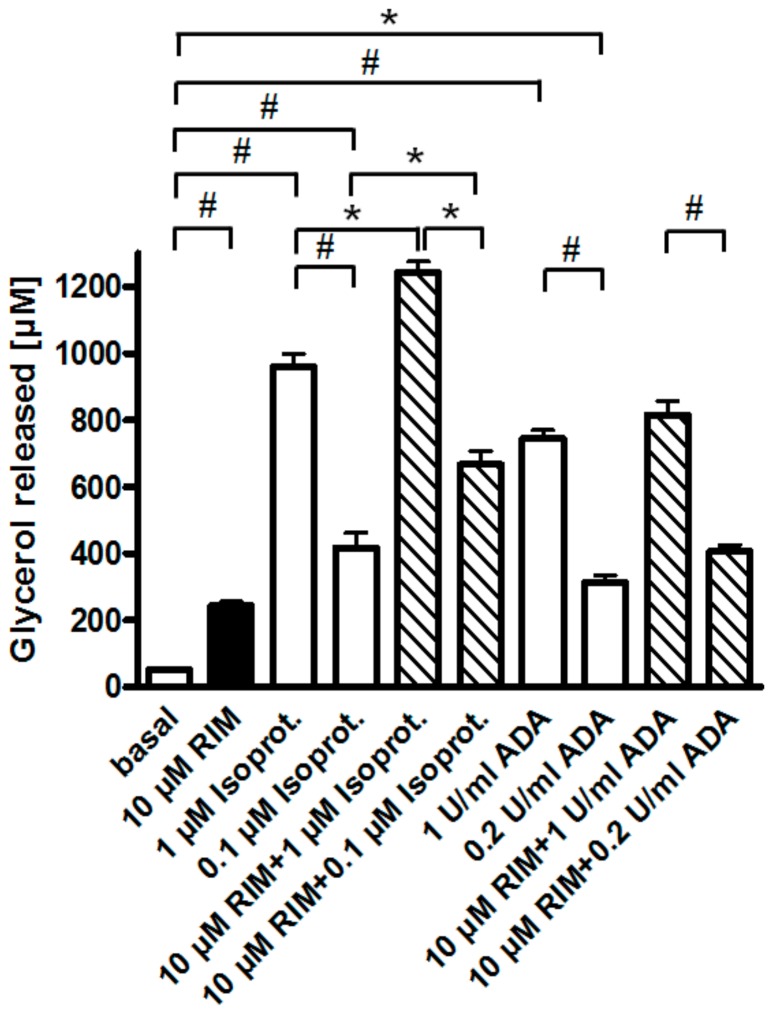
Effect of Rimonabant on the isoproterenol- and adenosine deaminase (ADA)-induced adipocyte lipolysis. Primary rat adipocytes were incubated (3 h, 37 °C) with half-maximally or maximally effective concentrations of isoproterenol (0.1, 1 µM) or ADA (0.2, 1 U/mL) or vehicle (basal) in the absence (open and filled bars) or presence (hatched bars) of Rimonabant (final concentration 10 µM). The concentration of glycerol released into the incubation medium was assayed. Mean ± SD of incubations in quadruplicate and measurements in duplicate. * *p* ≤ 0.05, ^#^ *p* ≤ 0.01; no significant differences for ADA between absence and presence of Rimonabant.

One possible explanation for the apparent upregulation of lipolysis through (partial) antagonism of the CB1R by Rimonabant is that, upon preparation of epididymal adipocytes from untreated young rats fed ad libitum, a portion of their CB1R remains specifically occupied by endocannabinoids, contributing to the almost complete suppression of lipolysis in basal adipocytes (see above). Consistent with negative regulation of adipocyte lipolysis by the CB1R via coupling to G_i/0_-proteins, glycerol and FA release induced by a maximally effective concentration of Rimonabant (10 µM) and half-maximally (0.2 U/mL) or maximally (1 U/mL) effective concentrations of adenosine deaminase (which leads to inactivation of the G_i_-coupled adenosine receptor) was subadditive and nonsignificant compared to the treatments alone (Figure 4). In contrast, isoproterenol, an agonist of the G_s_-coupled ß-adrenergic receptor, provoked significant and additive upregulation of lipolysis in combination with Rimonabant. In agreement with the latter finding, the ß-adrenergic receptor antagonist, propranolol, which concentration-dependently reduced the maximal isoproterenol-induced lipolysis to basal values, failed to block maximal stimulation of lipolysis by Rimonabant to any significant degree (Supplementary Figure S3).

### 2.3. Rimonabant Stimulates PKA (Protein Kinase A) in Adipocytes

**Figure 5:**
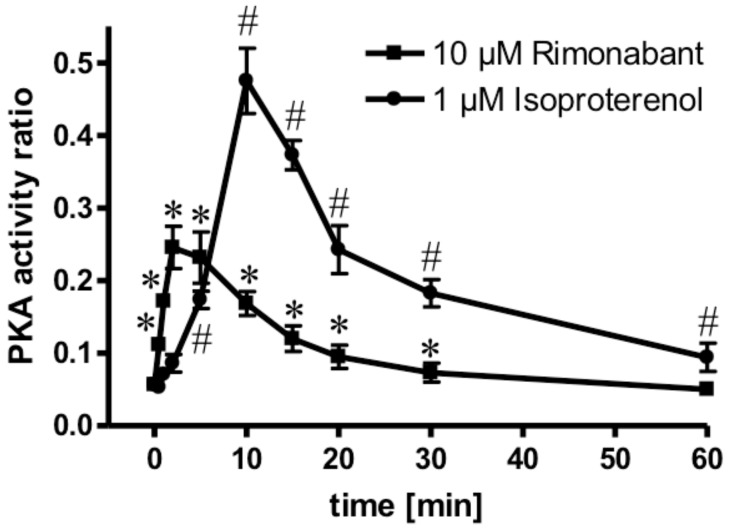
Activation of PKA by Rimonabant during adipocyte lipolysis. Primary rat adipocytes were incubated (37 °C) in the absence or presence of 1 µM isoproterenol (●) or 10 µM Rimonabant (■) for increasing periods of time and then rapidly deep-frozen. After preparation of cytosolic fractions, identical amounts of protein were assayed for the PKA activity ratio (see Materials and Methods). The portion of PKA activated at the time point of deep-freezing of the adipocytes was calculated as the ratio of Kemptide phosphorylated in the absence and presence of cAMP. Mean ± SD of three incubations with electrophoretic separations in duplicate. * (10 µM Rimonabant), # (1 µM Rimonabant) *p* ≤ 0.01 vs. time 0.

Next, the molecular mechanisms for the CB1R-dependent and independent Rimonabant-induced lipolysis in primary rat adipocytes were characterized. With regard to the former and considering previous [30] and the above results, which suggest the engagement of the CB1R/G_i/0_/adenylate cyclase-system, the effect of Rimonabant on the activation of PKA was studied. At maximally effective concentrations, Rimonabant as well as isoproterenol, a well-known activator of the (cyclic adenosine monophosphate (cAMP)/PKA pathway [22], led to considerable, but transient increases in the PKA activity ratio in primary rat adipocytes, albeit, with strikingly different time courses (Figure 5). For Rimonabant, the increase was characterized by a more rapid onset and shorter duration compared to that for isoproterenol. The lower PKA activity ratio at any time point upon challenge with Rimonabant vs. isoproterenol might explain the (approximately 80%) less pronounced stimulation of lipolysis by the former (see Figure 4).

Lipolysis elicited by a rise in cytosolic cAMP and activation of PKA in rat adipocytes is known to be inhibited by insulin with high sensitivity [22]. Insulin completely blocked the maximal isoproterenol-induced (1 µM) and half-maximal Rimonabant-induced (1 µM) lipolysis with comparable IC_50_ (0.03 and 0.09 nM, respectively) (Supplementary Figure S4). In contrast, lipolysis maximally stimulated by Rimonabant (10 µM) was reduced by insulin (> 1 nM) by only 40%–50%. This can be explained best by two distinct lipolytic mechanisms exerted by Rimonabant in primary rat adipocytes, which operate at concentrations below and above 1 µM and involve activation of PKA and its inhibition by insulin or not, respectively.

**Figure 6:**
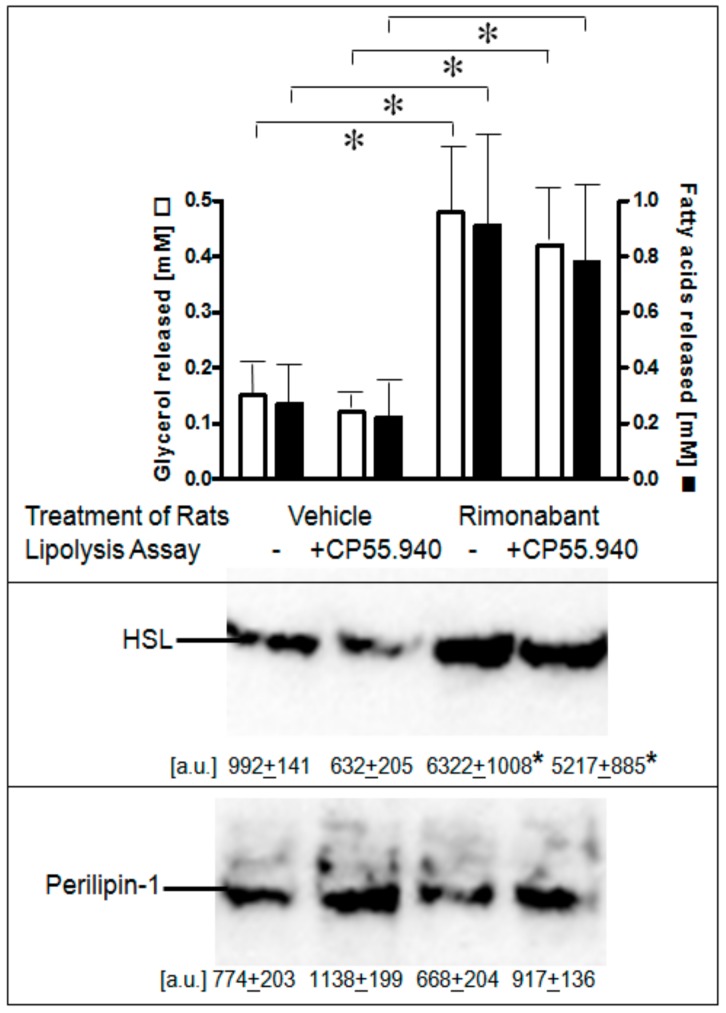
Stimulation of lipolysis in adipocytes from Rimonabant-treated rats. Male Wistar rats (280–320 g) were treated with one dose of Rimonabant (30 mg/kg, p.o.; RIM) or vehicle. After 5 h under free access to food, epididymal adipocytes were prepared by collagenase digestion and washed and then incubated (3 h, 37 °C) in the absence or presence of CP55.940 (10 µM final conc.). Upper panel: The amounts of glycerol and FA released into the incubation medium were determined enzymatically. Means of determinations in triplicate. Middle and lower panels: The amounts of HSL and perilipin-1 associated with LD, which were prepared from the homogenates of the adipocytes by centrifugation through sucrose cushions, were determined. For this, proteins contained in the top fraction were extracted from lipids, then separated by SDS-PAGE and analyzed for HSL and perilipin-1 by immunoblotting. The images of a typical experiment with electrophoretic runs in quadruplicate with their quantitative evaluations are shown. Mean ± SD of two different adipocyte/LD preparations with incubations/measurement in quadruplicate. * *p* ≤ 0.05; no significant differences between absence and presence of CP55.940.

Next, it was studied whether one or the other of the lipolytic activities of Rimonabant can be demonstrated in primary adipocytes isolated from normal fed rats which had been treated once with Rimonabant under conditions (i.e., single bolus of 30 mg/kg, p.o.) leading to acute short-term stimulation of lipolysis (Herling A.W., Müller G.A., Schmoll D.; unpublished data). Lipolysis was increased by 3- to 4-fold in adipocytes from Rimonabant- vs. vehicle-treated rats as reflected in both glycerol and FA release and was not significantly impaired by excess of CP55.940 present during the 3-h lipolysis assay in vitro (Figure 6, upper panel). Stimulation of lipolysis in adipocytes from Rimonabant-treated rats was well correlated to a 6- to 8-fold increased association of HSL with LD, which was not affected by the presence of CP55.940 (Figure 6, middle panel). The amounts of LD-associated perilipin-1 did not change significantly in response to Rimonabant (Figure 6, lower panel). It is difficult to reconcile the above finding that lipolysis induction by Rimonabant at 1 µM in rat adipocytes in vitro (see Figure 3) was inhibited by CP55.940 with the data on the persistence of lipolysis in adipocytes from the Rimonabant-treated rats in the presence of CP55.940. It may hint to operation in vivo of CB1R-independent lipolysis induction at the administered dose and/or to the failure of Rimonabant to exert direct CB1R-dependent lipolytic effects in adipocytes upon treatment of the rats. The differentiation between these possibilities would necessitate analysis of adipocyte lipolysis following treatment of rats with varying doses (below and above 30 mg/kg) of Rimonabant. Nevertheless, the animal studies unequivocally demonstrated lipolysis induction by Rimonabant in rats in vivo.

**Figure 7:**
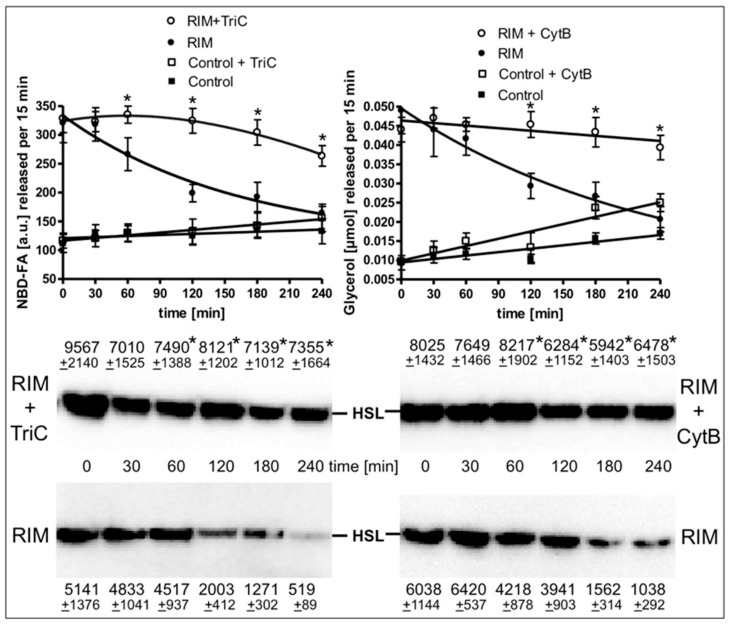
Loss of the Rimonabant-induced lipolysis and HSL translocation in adipocytes by ongoing lipid synthesis. Primary rat adipocytes, which had been metabolically labelled with -((7-nitrobenz-2-oxa-1,3-diazol-4-yl)amino)dodecanoic acid (NBD-FA), were pretreated (20 min, 37 °C) in the absence (Control) or presence of 10 µM Rimonabant (RIM). Thereafter, 500-µL aliquots of the incubation mixtures were transferred into 2-mL Eppendorf vials and then subjected to centrifugation (500x g, 2 min). Subsequently, the infranatant was removed by suction and the adipocytes were suspended in 2 mL of fresh incubation medium lacking Rimonabant. After two additional flotation cycles, the washed adipocytes were incubated (37 °C) in medium harboring 10 mM glucose and 0.1 nM insulin in the absence or presence of cytochalasin B (10 µM; CytB) or triacsin C (30 µM; TriC) for the periods indicated. Subsequently, the adipocytes were washed by flotation, resuspended and then incubated (15 min, 37 °C). Upper panel: The amounts of NBD-FA and glycerol released into the incubation medium were measured by fluorometry and enzymatically, respectively. Mean ± SD of two different adipocyte preparations with incubations/measurements in triplicate. Middle and lower panels: The amounts of HSL associated with LD, which were prepared from the homogenates of the adipocytes by centrifugation through sucrose cushions, were determined. For this, proteins contained in the top fraction were extracted from the lipids, then separated by SDS-PAGE and analyzed for HSL by immunoblotting. The images of a typical experiment with LD preparations and electrophoretic runs in triplicate with quantitative evaluations (mean ± SD) are shown. * *p* ≤ 0.05 for Rimonabant-treated adipocytes between absence and presence of TriC or CytB; no significant differences for control adipocytes.

The time course of the stimulation of lipolysis in adipocytes from Rimonabant-treated rats after their isolation and use in the lipolysis assay in vitro (Supplementary Figure S5) as well as in primary rat adipocytes, which had been metabolically labelled with 12-((7-nitrobenz-2-oxa-1,3-diazol-4-yl)amino)dodecanoic acid (NBD-FA) prior to their stimulation with Rimonabant and subsequent assay for lipolysis (Figure 7) was studied next. For this, the two populations of adipocytes were incubated for up to 4 h in the presence of high glucose and low insulin under conditions, which considerably favor lipid synthesis from glucose compared to lipolysis (Supplementary Table S1) and then studied for the release of glycerol, FA and NBD-FA. Lipolysis in adipocytes from Rimonabant-treated rats (Supplementary Figure S5) and in Rimonabant-incubated adipocytes (Figure 7, upper panel) was initially increased three- to fivefold compared to vehicle-treated animals and basal adipocytes, respectively. The lipolytic rates of both cell populations decreased with time reaching the values of the adipocytes from vehicle-treated animals and basal adipocytes, respectively. Surprisingly, the time-dependent loss of lipolysis in adipocytes from Rimonabant-treated rats (Supplementary Figure S5) as well as in Rimonabant-incubated adipocytes (Figure 7, upper panel) upon incubation in vitro was almost completely abrogated in the presence of inhibitors of acyl-CoA synthase, triacsin C, or glucose transport, cytochalasin B. Both inhibitors blocked lipid synthesis in primary rat adipocytes by 90% at least, but reduced isoproterenol-stimulated lipolysis only marginally (Supplementary Table S1). The reduced and constant glycerol/NBD-FA releases of Rimonabant-incubated adipocytes during the 4-h period of pronounced (absence of inhibitor) and blocked (presence of cytochalasin B or triacsin C) lipid synthesis, respectively, were correlated well to decreased and maintained association, respectively, of HSL with LD (Figure 7, middle and lower panels). These results argued that ongoing lipid synthesis and accompanying LD biogenesis apparently interfere with activation of lipolysis and translocation of HSL to LD in adipocytes by Rimonabant.

### 2.4. Rimonabant Increases Accessibility of LD for Lipolytic Cleavage in a Cell-Free System

The above experiments indicated that the observed stimulation of lipolysis by Rimonabant in primary rat adipocytes at concentrations above 1 µM and during prolonged incubation is not fully explained by binding to the CB1R and the resulting activation of PKA. In addition to PKA-dependent phosphorylation of HSL and perilipin-1 as the only unequivocal mechanism for upregulation of lipolysis by physiological stimuli in intact adipose cells [31,32,33], Okuda and coworkers provided evidence for a phosphorylation-independent direct increase in lipolysis upon incubation of a cell-free system with isoproterenol [34,35]. Consequently, the possibility of a direct interaction of Rimonabant with LD and/or HSL resulting in improved accessibility of the LD surface to and increased cleavage of triacylglycerol (TAG) by HSL was investigated. For this, NBD-FA-labelled LD were prepared from primary rat adipocytes. The relative purity of the isolated LD, which are formed by a lipid monolayer of phospholipids, cholesterol and FA (surface lipids, SL) with intercalated proteins [36] surrounding the LD core lipids (TAG, diacylglycerol, cholesteryl ester) like a shell, was confirmed by comparison of the lipid composition of LD with that of the adipocyte homogenate (Supplementary Table S2).

**Figure 8:**
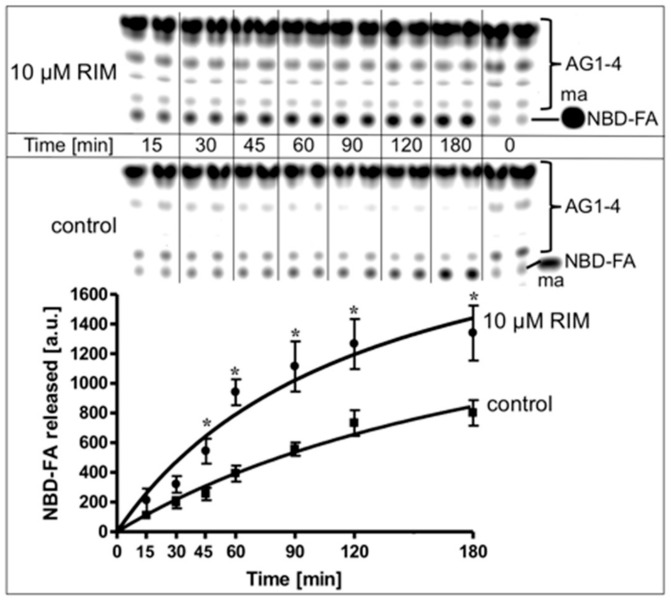
Stimulation of lipolysis in a cell-free system by Rimonabant. LD prepared from untreated rat adipocytes, which had been metabolically labelled with NBD-FA, were reconstituted with partially purified rat adipocyte HSL (see Materials and Methods) and then incubated (30 °C) for increasing periods in the absence (control) or presence of Rimonabant. The organic phase of the chloroform/methanol/HCl-extract of the total incubation mixture was analyzed by thin layer chromatography (TLC). The amount of NBD-FA lipolytically released from the NBD-FA-labelled acylglycerols (AG) 1–4 and run in parallel to a synthetic NBD-FA marker (ma) was quantitatively evaluated by fluorescence imaging. A fluorogram of a typical experiment with TLC runs in duplicate repeated two times is shown with quantitative evaluation (mean ± SD) of three independent incubations with TLC analysis in duplicate. * *p* ≤ 0.05 for LD incubated between absence and presence of Rimonabant.

For investigation of the effect of Rimonabant on lipolysis in a cell-free system reconstituted from native substrate and lipase, rat adipocyte LD, metabolically labelled with NBD-FA, were incubated with, partially purified rat adipocyte HSL in the absence or presence of Rimonabant and then assayed for the release of NBD-FA. In the control reaction, a moderate time-dependent release of NBD-FA was observed, arguing for productive interaction of HSL with LD. Rimonabant in the cell-free lipolysis system stimulated NBD-FA release twofold at 10 µM (Figure 8) and concentration-dependently up to threefold at 50 µM (EC_50_ = 15.8 µM) compared to control (Supplementary Figure S6A). This Rimonabant action in the cell-free system was not compromised by a 20-fold molar excess of CP55.490 (Supplementary Figure S6B).

Isoproterenol, but not PKA (in the presence of cAMP and ATP) and forskolin, also significantly increased NBD-FA release in the cell-free lipolysis system, albeit to a lower extent compared to isoproterenol (Supplementary Figure S6B). This was in agreement with previously published findings that part of the lipolytic activity of isoproterenol does not rely on PKA action, at least in vitro [35]. Compatible with a role of the phospholipids within the SL in down-regulating lipolysis [25,37,38], phospholipases (PL) of varying specificity caused a dramatic upregulation of NBD-FA release in the cell-free lipolysis system to up to the maximal Rimonabant effect, with phosphatidylcholine-specific PLC (PC-PLC) being most potent (Supplementary Figure S6A). Incubation of Rimonabant together with PL led to additive (phosphatidylcholine-specific PLD, PLD; phosphatidylinositol-specific PLC, PI-PLC), subadditive (PI-PLC) or no (PC-PLC) further upregulation of Rimonabant-induced cell-free lipolysis (Supplementary Figure S6A). Together these data suggest that PL as well as Rimonabant action facilitates the access of HSL to the LD surface. Disruption of the native structure of LD by detergent and ultrasonic treatment has also been reported to improve the efficiency of lipase catalysis, due to the increase in accessible surface area, the so-called interfacial activation [39,40]. To test whether these treatments substitute for Rimonabant or isoproterenol action, LD were emulsified in gum arabic and sonified before addition of HSL (Supplementary Figure S6C). As expected, this treatment led to NBD-FA release comparable with that of Rimonabant and isoproterenol acting at native LD. This indicated (i) that there is a maximal rate of lipolysis in the cell-free system which can be achieved with Rimonabant, PL action or interfacial activation and that (ii) structural integrity of the LD is required for Rimonabant stimulation of cell-free lipolysis. In agreement with the latter conclusion, in a typical HSL assay with radiolabelled trioleoylglycerol emulsified in a mixture of PC/PI and recombinant human HSL, Rimonabant up to 50 μM did not significantly stimulate lipolysis (Supplementary Figure S7).

**Figure 9:**
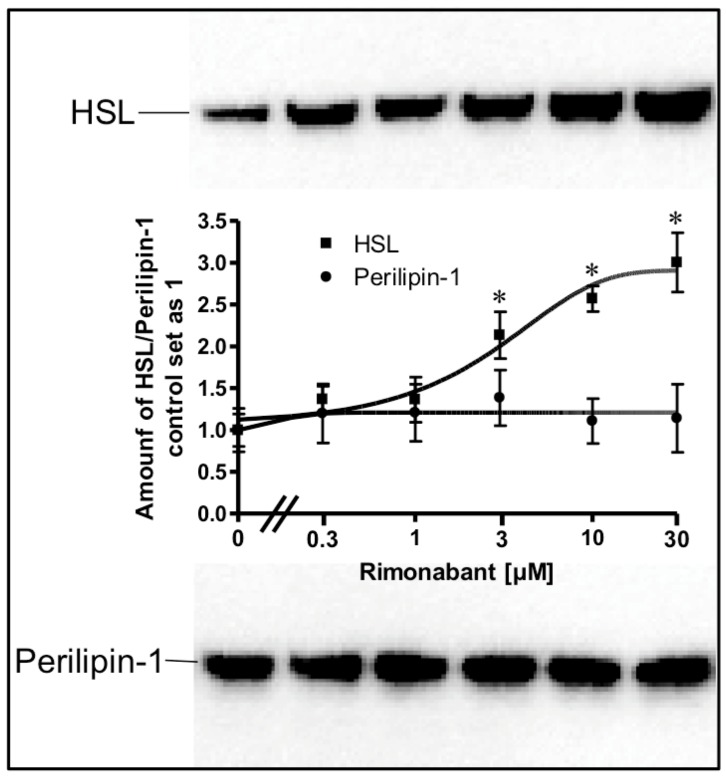
Increase in the amount of LD-associated HSL in a cell-free system by Rimonabant. Primary rat adipocytes were incubated (20 min, 37 °C) in the absence or presence of increasing concentrations of Rimonabant. Subsequently, LD were prepared from the washed adipocytes, reconstituted with partially purified rat adipocyte HSL and then incubated (90 min, 30 °C). LD were recovered from the total incubation mixtures by centrifugation through a sucrose cushion from the top fraction 6 (see Materials and Methods). Extracted proteins were separated by SDS-PAGE and analyzed for HSL (■) and perilipin-1 (●) by immunoblotting. The images of a typical experiment are shown repeated two times with similar results. The quantitative evaluation of the three experiments is also shown (mean ± SD). * *p* ≤ 0.05 for HSL recovered with LD between absence and presence of Rimonabant; no significant differences for perilipin-1.

To study the possibility that Rimonabant facilitates the access of HSL to LD by enhancing the interaction of HSL with LD and/or by removing perilipin-1 from LD, the amounts of LD-associated HSL and perilipin-1 were determined after sucrose gradient purification of the LD from the cell-free lipolysis system (Figure 9).

A considerable portion of HSL was recovered with LD already in the basal state, compatible with the relative distribution of HSL between LD and cytosol in unstimulated primary or cultured adipose cells, as previously reported in most but not all studies [24,27]. Incubation with Rimonabant led to a concentration-dependent increase in LD-associated HSL to up to threefold compared to basal (Figure 9). In contrast, the amount of perilipin-1 recovered with LD did not change with increasing concentrations of Rimonabant. These data suggested that Rimonabant treatment facilitates interaction of HSL with LD without causing concomitant detachment of perilipin-1.

### 2.5. Rimonabant Improves Accessibility of LD for Lipolytic Cleavage in Intact Adipocytes

**Figure 10:**
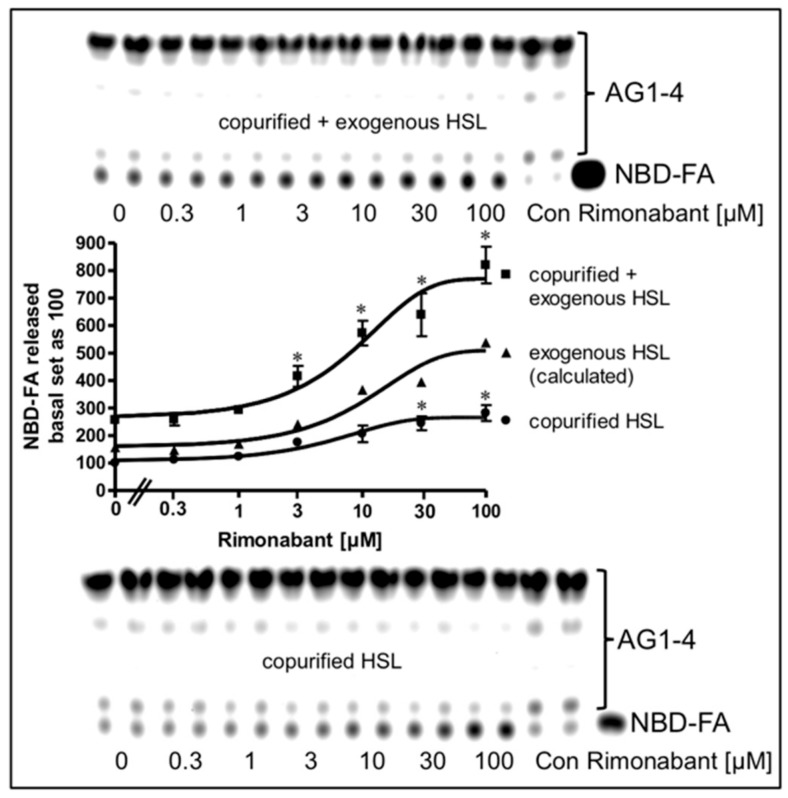
Stimulation of lipolysis in a cell-free system from Rimonabant-treated rat adipocytes. Primary rat adipocytes, which had been metabolically labelled with NBD-FA, were incubated (20 min, 37 °C) in the absence or presence of increasing concentrations of Rimonabant. Subsequently, LD were prepared from the washed adipocytes, reconstituted without (copurified HSL) or with partially purified rat adipocyte HSL (copurified + exogenous HSL)(see Materials and Methods) and then incubated (90 min, 30 °C). The organic phase of the chloroform/methanol/HCl-extract of the total incubation mixture was analyzed by TLC. The amount of NBD-FA lipolytically released from the NBD-FA-labelled AG1–4 and run in parallel to a synthetic NBD-FA marker (ma) was quantitatively evaluated by fluorescence imaging. Fluorogram of a typical experiment with TLC runs (only 1/5 of the volumes obtained from the total incubation mixtures of the “copurified + exogenous HSL” reactions were applied) in duplicate repeated two times; quantitative evaluation (mean ± SD) of the three different incubations with TLC analysis (corrected for the different volumes applied) in duplicate with ● calculated as difference (exogenous HSL) of the reaction containing and lacking exogenously added HSL. A blank value from a control reaction terminated immediately after the addition of HSL (copurified + exogenous HSL) or buffer (copurified HSL) was subtracted in each case. NBD-FA released in the absence of Rimonabant (basal) was set at 100 for each reaction containing or lacking exogenously added HSL. * *p* ≤ 0.05 for LD incubated with copurified plus exogenous HSL or copurified HSL alone between absence and presence of Rimonabant.

Next, the possibility was studied that the mechanism engaged by Rimonabant in the cell-free lipolysis system is also effective during CB1R-independent late-onset stimulation of lipolysis by Rimonabant in intact adipocytes. For this, LD were prepared from NBD-FA-labelled and then Rimonabant-treated primary rat adipocytes, and subsequently assayed for the release of NBD-FA in the cell-free lipolysis assay. In the absence of exogenously added HSL, a moderate amount of NBD-FA was released from acylglycerol (AG) 1–4 of untreated adipocytes (Figure 10, copurified HSL).

This intrinsic lipolysis was apparently due to HSL, remaining left at the LD during their preparation. This LD-associated HSL activity became significantly elevated upon challenge of the adipocytes with increasing concentrations of Rimonabant. Addition of partially purified rat HSL to the LD caused a 2.5-fold increase in NBD-FA release (Figure 10, copurified + exogenous HSL). Importantly, LD prepared from Rimonabant-treated adipocytes displayed more pronounced NBD-FA release compared to untreated LD with similar Rimonabant concentration dependence for the presence vs. absence of exogenous HSL, i.e., for the exogenous HSL alone (as difference between “exogenous + copurified” and “copurified” HSL). This strongly suggested that LD from Rimonabant-treated adipocytes represent better substrates for cleavage by exogenously added HSL than native LD. Interestingly, the same was true for LD from isoproterenol (1 µM)-treated (but not forskolin-treated) adipocytes, although the efficacy was very moderate compared to 50 µM Rimonabant (Supplementary Figure S8). As expected, lipolysis of the NBD-FA-labelled LD from Rimonabant (10 µM)-treated adipocytes by exogenously added HSL was not competed for by a 20-fold molar excess of CP55.940. This was in agreement with the absence of functional CB1R and downstream signaling to the lipolytic effectors in the cell-free lipolysis system. Addition of Rimonabant to the cell-free lipolysis system consisting of LD from adipocytes which had been treated with a submaximal (10 µM) or maximal (50 µM) concentration of Rimonabant and exogenously added HSL, led to further (and at 50 µM subadditive) increases in NBD-FA release (Supplementary Figure S8). These observations are compatible with identical mechanisms that trigger the improved accessibility of LD for binding of and degradation by HSL in intact adipocytes and in the cell-free lipolysis system in response to Rimonabant.

**Figure 11:**
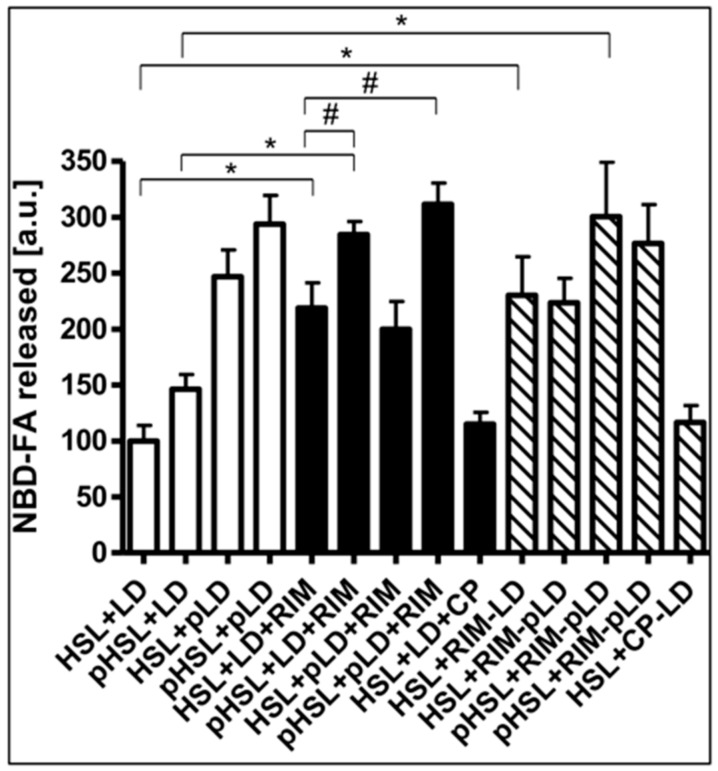
Effect of phosphorylation of HSL/LD on Rimonabant-induced lipolysis in the cell-free system. LD prepared from untreated (open, filled, hatched bars; LD) or Rimonabant (10 µM, 3 h, 37 °C)-treated (RIM-LD) or CP55.490-treated (50 µM; CP-LD) primary rat adipocytes, which had been metabolically labelled with NBD-FA, were incubated (30 min, 30 °C) with ATP in the presence (pLD) or absence (LD) of PKA catalytic subunit. Recombinant human HSL was incubated (30 min, 4 °C) with ATP in the presence (pHSL) or absence (HSL) of PKA catalytic subunit. After termination of the reactions and recovery of the LD and HSL, they were reconstituted as indicated and then incubated (90 min, 30 °C) in the presence or absence of 10 µM Rimonabant (RIM) or 50 µM CP55.490 (CP). After extraction of the total incubation mixtures with chloroform/methanol/HCl, the organic phases were analyzed by TLC. The amounts of NBD-FA lipolytically released from the NBD-FA-labelled AG1–4 were quantitatively evaluated by fluorescence imaging (mean ± SD of incubations in quadriplicate and TLC analysis in duplicate. NBD-FA released in the “HSL + LD” (absence of Rimonabant during cell-free lipolysis and adipocyte incubation, respectively) was set at 100. Values from corresponding reactions lacking exogenously added HSL were subtracted in each case in order to correct for HSL copurifying with the LD. Thus, the lipolysis activity depicted as NBD-FA released relies on the exogenously added recombinant human HSL rather than endogenous LD-associated rat HSL. * *p* ≤ 0.01, ^#^ *p* ≤ 0.05; significant differences are indicated only for physiologically relevant comparisons as discussed in the text.

Finally, the nature of the Rimonabant-induced increase in accessibility of LD for HSL was investigated. For this, the effects of phosphorylation of recombinant human HSL and LD by PKA in vitro on the Rimonabant stimulation of NBD-FA release from NBD-FA-labelled LD as well as of HSL and perilipin-1 translocation to and from LD, respectively, during cell-free lipolysis were analyzed. The efficacy of the phosphorylation of HSL and LD from untreated as well as Rimonabant-treated adipocytes was confirmed by the considerable incorporation of ^32^P_i_ into total precipitable proteins of the various incubation mixtures, which was dependent on the presence of PKA catalytic subunit and absence of a specific PKA inhibitor peptide (Supplementary Table S3). Phosphorylation of recombinant human HSL increased cell-free lipolysis by less than 50%, only, whereas phosphorylated LD stimulated lipolysis by around 2.5-fold, irrespective of the phosphorylation status of HSL, compared to the unphosphorylated counterparts (Figure 11, open bars). Rimonabant did not significantly affect phosphorylation of HSL and LD by PKA, but led to twofold higher lipolysis vs. control with unphosphorylated HSL and LD. This was further increased to up to threefold by the use of phosphorylated HSL, but not phosphorylated LD (Figure 11, filled bars).

In contrast, CP55.490 was ineffective. Similarly, LD prepared from Rimonabant-treated, but not from CP-55.490-treated adipocytes, stimulated lipolysis by 2.2-fold in the presence of unphosphorylated HSL and by 2.8-fold in the presence of phosphorylated HSL (Figure 11, hatched bars). The phosphorylation state of the LD did not affect cell-free lipolysis in response to Rimonabant. Thus, the presence of Rimonabant or of LD from Rimonabant-treated adipocytes in the cell-free system seems to substitute for the PKA-dependent phosphorylation of LD in causing a twofold stimulation of lipolysis, but fails to substitute for the moderate stimulation of lipolysis by phosphorylated HSL.

The stimulation of cell-free lipolysis by LD prepared from Rimonabant-treated adipocytes was strictly dependent on the incubation time with Rimonabant, but completely unaffected by the simultaneous presence of CP55.490, which did not increase basal lipolysis per se (Supplementary Figure S9). The time course for upregulation of the cell-free lipolysis by LD from Rimonabant-treated cells closely resembled that for Rimonabant stimulation of lipolysis in intact adipocytes in the presence of CP55.490 (see Supplementary Figure S2). The association of HSL with LD was stimulated by the use of phosphorylated HSL or phosphorylated LD or LD from Rimonabant-treated adipocytes as well as by the presence of Rimonabant during the cell-free lipolysis or by combinations thereof to around 2- to 2.5-fold in each case in comparison to the unphosphorylated/untreated counterparts (Supplementary Table S3). Thus, PKA-dependent phosphorylation of LD or HSL as well as the corresponding mechanism engaged by Rimonabant seem to be sufficient and rate-limiting for the association of HSL with LD and the accompanying stimulation of lipolysis. It should be noted, that this analysis addressed the association of exogenous recombinant human HSL, exclusively, due to the species specificity (rat vs. human) of the antibodies used for immunoblotting. Changes in the amount of perilipin-1 associated with LD were not detected under either condition (data not shown). This confirmed the above findings that stimulation of lipolysis in the cell-free system relies on the translocation of HSL to rather than of perilipin-1 from LD, irrespective of whether being driven by phosphorylation of HSL or LD or by the Rimonabant-induced mechanism.

## 3. Discussion

The present study addresses the mechanisms underlying the observed acute and transient stimulation of lipolysis by Rimonabant in fed Wistar rats, as reflected in the rapid-onset, moderate, short-lasting and dose-dependent increase in serum FA levels. For this, it was studied whether Rimonabant exerts direct effects on primary rat adipocytes, i.e., upon incubation in culture, and, if so, whether they are mediated by the peripheral, i.e., adipocyte CB1R or by alternative mechanisms.

### 3.1. The Direct CB1R-Dependent Lipolytic Activity of Rimonabant

Rimonabant, was found to stimulate lipolysis in primary rat adipocytes upon incubation at low µM-concentrations by up to 25% of the maximal isoproterenol effect (Figure 1) as well as in fed normal rats upon single administration of 30 mg/kg (Figure 6). Subsequent detailed analysis revealed two distinct lipolytic activities, one dependent on, the other one independent of CB1R. The CB1R-dependent lipolytic mechanism requires concentrations of 1–10 µM and accounts for about 50% of the total lipolytic activity of Rimonabant. It relies on activation of PKA and translocation of HSL to and perilipin-1 from the LD. Most likely, G_i/0_-coupled CB1R is occupied by endocannabinoids in primary rat adipocytes and undergoes antagonism and deinhibition by excess of Rimonabant. This would explain the lack of inhibition of basal (= absence of Rimonabant) lipolysis by exogenous CB1R agonist (Figure 3). Interestingly, adipose tissue is able to accumulate lipophilic cannabinoid(-like) substances and expresses 2-monoacylglycerol lipase, which plays a key role in the synthesis of endocannabinoids [7]. The reduction in the CB1R-dependent lipolytic activity of Rimonabant in adipocytes derived from Rimonabant-treated rats may rely on (i) loss of CB1R occupancy, (ii) loss of CB1R downstream signaling to the lipolysis machinery or (iii) feedback inhibition of the cAMP-PKA-HSL/perilipin-1 signaling pathway as a consequence of sustained (CB1R-dependent and independent) lipolysis activation during the period after Rimonabant administration before preparation of the adipocytes.

Interestingly, CB1R antagonists have been observed to act as inverse agonists independently of elevated endocannabinoid levels, at least in vitro [41]. This could also explain lipolysis stimulation by Rimonabant. Alternatively, G protein-coupled receptors different from CB1R and of medium to low affinity for Rimonabant may be expressed in rat adipocytes and negatively regulate lipolysis. They would explain the seemingly high effective Rimonabant concentrations required that exceed the K_d_ for the brain CB1R by 100- to 1000-fold. Natural and synthetic cannabinoids have been demonstrated to elicit a variety of effects in peripheral cells, such as vasodilatation in vascular endothelial cells via protein kinase B [42] and depolarization in epithelial cells via Ca^2+^-channels [43]. Those ligands bind to non-CB1/2 G protein-coupled receptors with roughly 10- to 100-fold lower affinity compared to CB1R [44]. Adipocyte lipolysis is known to be regulated by protein kinase B- and Ca^2+^-dependent mechanisms [34,45] which represent routes how lipolysis may be regulated by the endocannabinoid system. Furthermore, the cross-talk between the cAMP-PKA-HSL/perilipin-1 axis and the endocannabinoid system could also involve adenosine monophosphate-dependent protein kinase (AMPK). Interestingly, AMPK undergoes inhibition in both adipose tissue and liver of rats upon intraperitoneal injection of a CB1R agonist [46]. This finding suggests that antagonism of CB1R leads to phosphorylation and activation of AMPK. However, the consequence of the reported direct phosphorylation of HSL by AMPK on adipocyte lipolysis has been a subject of controversy so far [47,48].

### 3.2. The Direct CB1R-Independent Lipolytic Activity of Rimonabant

Exposure of adipocytes to Rimonabant at higher concentrations (>10 µM) and for longer periods stimulates lipolysis by a mechanism which does not depend on functional CB1R or other receptors coupled to the cAMP–PKA axis. Strikingly, incubation of native LD prepared from primary rat adipocytes, but not of synthetic emulsified neutral lipids, with Rimonabant increases the affinity of the LD for association with and degradation by HSL. The mechanism engaged by Rimonabant in cell-free lipolysis or in LD prepared from Rimonabant-treated adipocytes is apparently mimicked by PKA-dependent phosphorylation of LD proteins rather than HSL (Supplementary Figure S9). This putative primary site of Rimonabant action at LD is compatible with the current view of the crucial role of LD-associated proteins in the regulation of lipolysis [49,50]. A protein coat formed by unique polypeptides, such as the perilipin family, seems to be responsible for the translocation to and anchorage of HSL at the LD surface, as well as for the protection of the LD core lipids from unlimited lipolytic digestion [49,50]. Phosphorylation of perilipin-1 by PKA has previously been shown to support lipolysis [48,50]. It is conceivable that Rimonabant somehow substitutes for this role of PKA-phosphorylated perilipin-1 or other LD proteins by spontaneous intercalation into the LD surface. This may cause structural rearrangements that ultimately lead to improved association of HSL with LD, as demonstrated in the cell-free system (Figure 9).

Most lipases gain access to their substrate lipids without the aid of substrate-associated proteins during a process termed interfacial activation [39,40]. This explains why the activity of lipases is enhanced on insoluble substrates (such as emulsions) in comparison with the same substrates in truly monomeric solutions. 3-D structures of lipases suggest that the interfacial activation process might be due to the presence of the lid domain covering the active site of many lipases in solution [51,52]. Upon contact with a lipid–water interface (during formation of an emulsion), a conformational rearrangement might result in opening of this lid, making the active site accessible. Thus, it is possible that opening of this lid during interfacial activation of HSL in course of its translocation to the LD becomes facilitated by Rimonabant molecules intercalated into the LD SL. However, this mechanism is not compatible with (i) the failure of Rimonabant to stimulate lipolytic cleavage of synthetic lipids emulsified with phospholipids (Supplementary Figure S7), (ii) the requirement of native LD and of absence of emulsifiers for Rimonabant stimulation of lipolysis in the cell-free system (Supplementary Figure S6A,C) and (iii) the parallel increases in the association of HSL with LD and lipolytic activity in the cell-free system (Supplementary Figure S9A,C). In addition, detailed kinetic analysis of HSL action on short- and long-chain substrates in the presence of a covalent inhibitor raised doubts whether typical lid domain is expressed in HSL [21]. Taken together, interference with the complex LD surface of rat adipocytes seems to represent the most likely mechanism underlying the CB1R-independent induction of the association of HSL with LD in response to Rimonabant. The receptor-independent lipolysis stimulation by isoproterenol (see Supplementary Figure S6A, [34,35]) may have a similar mechanistic basis, although Rimonabant and isoproterenol do not share any structural features. Together with previous findings, such as those obtained with an insect cell-free system [53], the present data argue for a mechanistic link between lipolysis activation and structural/functional alterations of the LD protein coat. Those can be exerted by PKA-dependent phosphorylation of LD proteins or a Rimonabant-induced mechanism, which is possibly based on the direct interaction of Rimonabant with the LD surface.

### 3.3. Physiological Relevance of the Direct Lipolytic Activity of Rimonabant

Importantly, accumulation of Rimonabant in adipose cells on the basis of its lipophilic structure has been revealed in pharmacokinetic studies with rodents, leading to concentrations at the plasma membranes and/or LD surface of adipocytes (>1 and >10 µM, respectively). Those exceed the C_max_ of Rimonabant in the serum of treated mice by two to three orders of magnitude and may be sufficient for initiation of its direct CB1R-dependent and/or independent lipolytic activity. Nevertheless, the direct CB1R-independent lipolytic activity of Rimonabant as detectable in vitro might be of minor importance in vivo since (i) Rimonabant has been shown to exert no significant effects on body weight gain, adipose tissue mass and cellular adiposity in CB1R knockout mice fed a high-fat diet, which are lean and resistant to diet-induced obesity [54], (ii) the transcriptional patterns in white adipose tissue and lipolysis stimulation are similar between Rimonabant-treated obese and high fat diet-fed CB1R knockout mice [55] and (iii) other known direct Rimonabant effects on adipocytes, as manifested in the induction of mRNA and protein expression, as well as in the secretion of adiponectin in cultured mouse 3T3 F442A adipocytes, were recapitulated in the adipose tissue of Rimonabant-treated wild type, but not CB1R knockout mice [28]. Thus, the available animal data favour a CB1R-mediated mechanism for the upregulation of adipocyte lipolysis during acute Rimonabant treatment, such as nervous stimulation in response to antagonism of hypothalamic CB1R, albeit a comparative analysis of serum FA levels between Rimonabant-treated wild type and CB1R knockout mice is still missing. Furthermore, it cannot be excluded that the observed acute minor Rimonabant-induced increase of FA in the serum of anaesthetized fed Wistar rats (Herling A.W., unpublished data) is due to adipose tissue lipolysis, induced by Rimonabant in adipocyte-autonomous manner, exclusively, rather than via the hypothalamic axis.

Finally, it is tempting to speculate that the direct effect of Rimonabant on lipolysis is not restricted to adipose tissue. HSL is almost ubiquitously expressed, and regulated mobilization of endogenous fat stores is crucial for many cell types [33]. Interestingly, the Rimonabant-induced elevated levels in serum FA of fed rats were found to be associated with increased levels of intramyocellular lipids for 6 h after a single administration of Rimonabant, which subsequently declined to levels lower than those of pair-fed control animals at 20 h [20]. This release of FA from intramyocellular lipids for subsequent FA oxidation in muscle tissues could be based on the stimulation of myocyte lipolysis in myocyte-autonomous manner by CB1R-dependent and/or independent mechanisms, similar to those shown to be effective in rat adipocytes in this study.

The CB1R-dependent and independent mechanisms engaged by the CB1R antagonist Rimonabant and operating directly at the adipocytes for stimulation of lipolysis, as reported here, may be of potential value for the future identification of novel targets and drug candidates for anti-obesity/diabetes therapy on basis of the pharmacological aim of lipolysis stimulation. Addressing the CB1R and/ or LD at adipose tissue, exclusively, has the crucial advantage of bypassing putative psychiatric side effects in course of engagement of hypothalamic CB1R. In fact, a novel potent CB1R antagonist, which has a low brain-to-plasma concentration distribution and does not exhibit centrally mediated neurobehavioral effects, was recently found to trigger significant weight loss and improvement of glycemic control, insulin sensitivity and adipose tissue inflammation in the absence of relevant reduction of food intake [55]. In addition, a different and strictly peripheral CB1R antagonist was demonstrated to increase the expression of genes involved in brown adipose tissue thermogenesis, to decrease the LD size in brown adipocytes and to enhance uncoupled respiration [19]. These therapeutically beneficial effects are best explained by direct CB1R-dependent or independent action of these (peripherally residing) CB1R antagonists at adipocytes.

Moreover, a multitude of novel Rimonabant structural analogs and hybrid compounds has been synthesized during the last years by Adriano Mollica’s and other groups for the therapy of obesity, inflammatory diseases, analgesia, edema and depression, such as Rimonabant-opioid peptide hybrids [56], Rimonabant–Fubinaca hybrids [57] and Rimonabant-indazole scaffold hybrids [58]. It will be interesting to investigate whether those Rimonabant derivatives, designed for (full, partial or inverse) agonism and antagonism towards brain CB1/2R, also manage to engage CB1R-dependent or independent mechanisms in cell-autonomous manner, e.g., at adipose or other relevant tissues. This would emphasize the potential of certain Rimonabant derivatives to act as drug molecules without the need to tackle the central nervous system, in general, and CB1/2R, in particular.

## 4. Materials and Methods

### 4.1. Animal Handling and Rimonabant Treatment

Male Wistar rats (Crl:WI(WU) were obtained from Charles River (Sulzfeld, Germany). They were housed two per cage in an environmentally controlled room with a 12:12-h light–dark circle (light on at 06:00) and ad libitum access to food and water. Standard rat chow (17.7 kJ/g, Ssniff diet R/M-H, V1535 with 18% crude protein, 4.7% sugar, and 3.5% crude fat; Ssniff, Soest, Germany) was used for 14 weeks. Thereafter, populations of 12 rats (280–320 g) were treated (5 h) with a single bolus administration of Rimonabant (30 mg/kg, p.o.) or vehicle, each, under free access to food. Following terminal isoflurane anesthesia, epididymal adipocytes were prepared by collagenase digestion. All experimental procedures were conducted in accordance with the German Animal Protection Law (paragraph 6) and corresponded to international animal welfare legislation and rules.

### 4.2. Preparation and Incubation of Primary Rat Adipocytes

Rat adipocytes were isolated by performing collagenase digestion of epididymal fat pads from untreated male Wistar rats (140–160 g, fed with water and standard laboratory diet ad libitum) or Rimonabant-/vehicle-treated rats, see above), followed by incubation in primary culture with DMEM in the presence of 25 mM glucose and 2% bovine serum albumin (BSA) [59]. Adipocytes were analysed for the release of glycerol and endogenous or NBD-FA-labelled fatty acids into the incubation medium [60] as described previously. Rimonabant (Sanofi-Aventis), CB1R agonists CP55.940 and WIN55.212-2 (Tocris) and inverse agonists AM281 and AM251, CB2R inverse agonist AM630, forskolin, isobutylmethylxanthine (IBMX), isoproterenol and propranolol (all Sigma-Aldrich, Deisenhofen, Germany) were dissolved at 10 mM as dimethylsulphoxide (DMSO) stock solution. For the treatment of adipocytes or myocytes, Rimonabant or CP55.940 was added directly from the stock solution to the incubation medium containing the cells and BSA (see above) without prior dilution. For incubation of the cell-free lipolysis system, Rimonabant or CP55.940 was added directly from the stock solution to the reaction mixture containing the LD and BSA (see below) without prior dilution. The final DMSO concentration during (ligand or control) incubation of cells and LD was kept constant at 1%. Metabolic labeling of the AG of isolated rat adipocytes was achieved by incubation (60 min, 37 °C) with the fluorescent fatty acid derivative, NBD-FA (0.5 mM, coupled to BSA), in the presence of 1 nM human insulin, as outlined recently [61].

### 4.3. Protein and Lipid Composition of LD from Adipocytes

LD were prepared according to Greenberg and coworkers [62] and adaptions from Wolins and coworkers [63] with the following modifications: After the incubation, the adipocytes were washed two times with Krebs-Ringer-Henseleit buffer by flotation and removal of the infranatant by suction and then homogenized in 25 mM Tris-HCl, pH 7.4, 250 mM sucrose, 5 mM NaF, 10 mM NaPP_i_, 1 mM Na_3_VO_4_, 1 mM ethylene diamine tetraacetic acid (EDTA), 20 µg/mL leupeptin, 1 mM benzamidine, 0.5 mM phenylmethylsulfonylfluoride (PMSF) at 15 °C. After centrifugation (1000× g, 5 min, 15 °C), 1 mL of the supernatant was combined with 1.5 mL of 65% sucrose (w/v) and poured into a 5-mL centrifuge tube. 1.5 mL of 10% sucrose (w/v) was then layered on top of the sucrose cushion. The tube was filled to capacity with buffer A. The gradient was centrifuged (172,000× *g*, 60 min, 15 °C) and then allowed to coast to rest. The floating fraction of LD was visualized as the upper white layer of the gradient, and was isolated by suction with a syringe (0.8 mL). After one washing cycle with buffer A, the LD were suspended in an equal volume of 4-fold sample buffer containing 10% SDS by incubation (10 min, 65 °C) and treatment in a bath sonicator (10 min, 25 °C, max. power) for extraction of proteins. After centrifugation (10,000× g, 5 min, 25 °C), proteins contained in the infranatant were separated by SDS-PAGE. Alternatively, the gradient was fractionated from bottom to top by suction with a syringe (0.8 mL each). Proteins of fractions 1–5 were precipitated with 5% trichloroacetic acid (TCA) (15 min, 2 °C, 15,000× g, 10 min), washed three times with ice-cold acetone, dried and finally suspended in sample buffer. Fraction 6 was extracted for protein (see above). All samples were analysed by SDS-PAGE and immunoblotting. The following antibodies were used; anti-HSL (1:750) raised in rabbits using a synthetic peptide; GPRLELRPRPQQAPRS, corresponding to rat HSL amino acids 326–341 [64]; anti-perilipin-1 (1:2000) raised in guinea pig against a synthetic peptide corresponding to the N-terminal region of human perilipin A and B (GP29, Progen Biotechnik, Heidelberg, Germany); anti-S3-12 (1:500) raised in rabbits against the N-terminal region of rat S3–12; anti-GRP78 (1:1000); anti-calnexin (1:500) raised in rabbits (Stressgen Biotechnologies Corp., Victoria, Canada); anti-caveolin-1 (1:2000) raised in rabbits (BD Transduction Laboratories, San Jose, CA); anti-adipophilin raised in guinea pig (Research Diagnostics Inc., Flanders, NJ); anti-TIP47 raised in rabbits against a synthetic peptide, MSSNGTDAPAEAQAAMEEPVC.

### 4.4. Cell-Free Lipolysis System

LD were prepared from isolated rat adipocytes, which had been metabolically labelled with NBD-FA, by suspending 1 mL of washed and packed cells in 5 mL of buffer C (50 mM Tris-HCl, pH 7.4, 250 mM sucrose, 1 mM EDTA, 500 mM NaCl, 5 mM KCl, 1 mM MgCl_2_, 2% BSA, 1 mM DTT, 0.1 mM benzamidine, 10 µg/mL leupeptin, 1 µg/mL aprotinin) containing 0.005% octylglucoside, followed by homogenization with a Potter–Elvehjem glass homogenizer with a loosely fitting Teflon pestle. The homogenate was overlaid with 2 mL of buffer C lacking sucrose and centrifuged (100,000× g, 1 h, 4 °C). LD were recovered from the fat cake at the top of the tube and purified from adherent cytosolic components by suspending in 6 mL of buffer C containing 20% (w/v) sucrose. A 2-mL cushion of buffer C lacking sucrose was laid on top. After centrifugation (100,000× g, 1 h, 4 °C), the LD were collected from the top and resuspended in 0.5 mL of buffer C lacking sucrose. Cell-free lipolysis was performed in glass tubes as described previously [34,35] with the following modifications: 40 µL of packed NBD-FA-labelled LD were incubated (90 min, 30 °C, constant shaking) with 80 µL of HSL-solution (100–200 µg protein) and 120 µL of buffer C containing 2% BSA, 2 mM DTT with or without 4.2 mg gum arabic. For incubation of the cell-free lipolysis system, Rimonabant or CP55.940 was added directly from the 50-mM stock solution to the reaction mixture containing the LD without prior dilution. The final DMSO concentration during all incubations was kept constant at 0.1%. Portions of the lipolysis reaction were terminated by extraction of the total incubation mixture with 720 µL of chloroform/methanol/0.1 N HCl (3/1/1, by vol.). After vortexing and centrifugation (2000× g, 2 min) of the mixture, the organic phase was collected, dried under a stream of N_2_ and resuspended in 40 µL of tetrahydrofurane. 10-µL portions were analyzed by TLC (Silica Gel G plates, Merck Darmstadt, Germany; diethylether/petrolether/acetic acid, 78/22/1 by vol.). NBD-FA released from the total NBD-FA-labelled AG was quantitatively evaluated by phosphorimaging (Storm 860, Molecular Dynamics, Krefeld, Germany) by using imaging software (ImageQuant, Molecular Dynamics), as described previously [65]. A blank value of a control reaction terminated immediately after addition of HSL-solution to the LD was subtracted in each case. Other portions of the lipolysis reaction were supplemented with 760 µL of buffer C containing protease inhibitors and centrifuged through sucrose cushions for the recovery of LD, which were then analyzed for protein composition (see above).

### 4.5. Phosphorylation of LD and HSL In Vitro

LD (40 µL) and/or recombinant human HSL (1–3 µg) were incubated (30 min, 30/4 °C) in 200 µL of kinase buffer (50 mM MOPS, 2 mM magnesium acetate, 1 mM EDTA, 2 mM DTT, 5 mM NaF, 2 mM imidazole, 10 mM glycerolphosphate), PKA catalytic subunit (0.8 units) and 0.5 mM ATP. After termination of the reaction by the addition of 20 µL of 50 mM EDTA, the LD were collected by centrifugation (2000× g, 2 min) from the top and washed once with buffer C and finally resuspended in 40 µL of buffer C for use in the cell-free lipolysis system. HSL was recovered from the infranatant and concentrated by absorption and elution from Ni^+^-columns as previously described for the purification [66]. Alternatively, for measurement of the phosphorylation reaction, 0.1 mM ATP containing 0.2 µCi [γ-^32^P]ATP was used. After termination of the reaction, the LD-associated proteins and/or HSL were precipitated (–20 °C, overnight) from the total incubation mixtures with acetone (80%, by vol.) and TCA (10%, by vol.), then collected by centrifugation (15,000× g, 15 min, 4 °C), washed with ice-cold acetone (100%, by vol.) and finally dissolved in 100 µL of 0.1 N NaOH for determination of radioactivity (in 10 mL of liquid scintillation cocktail).

### 4.6. Lipid Analysis

Total adipocyte homogenates or LD were extracted with *n*-hexane/2-propanol (3/2, by vol.). The lipid extracts were separated by TLC (Silica Gel G; petroleum ether/diethyl ether/methanol/acetic acid, 90/7/2/0.5) with oleic acid, dioleine (sn-1,2), trioleine, cholesteryl oleate and dipalmitoyl phosphatidylcholine (10 µg/mL each) run in parallel and visualized with iodine vapor as standards. Total cholesterol, FA, TAG and cholesteryl ester content were determined from the correspondingly separated and eluted TLC fractions comigrating with the standards as described previously [67]. Total phospholipid content was determined by digesting (1 h, 180 °C) the phospholipid fraction in deionized water and perchloric acid followed by the addition of ammonium molybdate and ascorbic acid [68]. The sample was further heated (5 min, 100 °C). After cooling, the absorbance was measured at 797 and 660 nm for evaluation of total phosphorus. The values were normalized for the total protein content of each sample measured in the dried protein extract.

### 4.7. PKA Activity Ratio

The incubation of the adipocytes was terminated by rapid deep-freezing (liquid N_2_) of the total reaction mixture. After thawing, centrifugation (15,000× g, 2 min, 4 °C), and addition of the same volume of two-fold kinase buffer (50 mM Tris-HCl, pH 7.4, 10 mM NaF, 20 mM NaPP_i_, 20 µg/mL leupeptin, 2 mM benzamidine, 0.2 mM PMSF, 200 µM IBMX, 2 mM MgCl_2_) to the supernatant, PKA activity was measured by incubation (5 min, 25 °C) of identical amounts of cytosolic protein with 0.5 mM ATP in the absence or presence of 1 µM cAMP as phosphorylation of rhodamine-labelled substrate peptide, Kemptide, according to published procedures [69]. Phosphorylated and unphosphorylated Kemptide were separated by agarose gel electrophoresis and quantitatively evaluated by fluorescence imaging.

### 4.8. Statistics

Analysis and fitting of primary data (concentration-response curves) were performed with FitMaster Origin-based software (Origin). Statistical significance was calculated using GraphPad Prism6 software (version 6.0.2, GraphPad Software) on the basis of three different tests according to the experimental design as follows: One-tailed unpaired *t*-test (Figure 1, Figure 2, Figure 6; Supplementary Table S1), two-tailed unpaired *t*-test (Figure 7, Figure 8, Figure 11; Supplementary Figures S2, S5, S7, S9; Supplementary Table S3), one-way ANOVA performed with Tukey’s multiple comparison post-test (Figure 3, Figure 4, Figure 5, Figure 9, Figure 10; Supplementary Figures S3, S4, S6, S8).

### 4.9. Miscellaneous

NBD-FA [64] and Rimonabant [70] were synthesized as described previously. Glycerol and FA were determined enzymatically, as outlined recently [60]. Lipid synthesis in isolated rat adipocytes was measured as the incorporation of [^3^H]glucose into total toluene-soluble ^3^H-labelled AG as reported previously [71]. Partially purified rat HSL-solution was prepared from the cytosol of untreated rat adipocytes in buffer C containing 1 mM DTT, 5 µg/mL leupeptin, 20 µg/mL aprotinin, 1 mM benzamidine, and deprived of lipoprotein lipase by passing through a heparin-Sepharose column as pH 5.2-precipitate (1 M NaCl did not significantly suppress TAG cleavage activity indicating almost quantitative removal of lipoprotein lipase), as published previously [66,72]. Purification of recombinant human His-tagged HSL and the HSL activity assay with [^3^H]trioleoylglycerol emulsified with phosphatidylcholine (PC) and phosphatidylinositol (PI) by ultrasonic treatment (5 min, ultrasonic bath incubator, full power) were carried out, as described previously [21,66]. Protein concentration was determined by using the BCA method (Pierce) with BSA as calibration standard. SDS-PAGE (Novex 8%–14%, Tris-glycine, precast gels with morpholinopropanesulfonic acid-SDS running buffer), immunoblotting by using polyvinylidene difluoride membranes and subsequent chemiluminescent detection (ECL, Amersham-Buchler) and quantitative evaluation of the lumi images (Roche Lumiimager and software) were carried out as reported earlier [65], unless indicated otherwise.

## Supplementary Materials

The following are available online at https://www.mdpi.com/1420-3049/25/4/896/s1, Supplementary Figure S1: Effect of Rimonabant on the protein and lipid composition of LD, Supplementary Figure S2: Time course of the Rimonabant-induced adipocyte lipolysis, Supplementary Figure S3: Differential effect of propranolol on the isoproterenol- and Rimonabant-induced adipocyte lipolysis, Supplementary Figure S4: Partial inhibition of Rimonabant-induced adipocyte lipolysis by insulin, Supplementary Figure S5: Stimulation of lipolysis in adipocytes from Rimonabant-treated rats, Supplementary Figure S6: Stimulation of lipolysis in a cell-free system by Rimonabant, Supplementary Figure S7: Effect of Rimonabant on HSL activity *in vitro*, Supplementary Figure S8: Stimulation of lipolysis in a cell-free system from Rimonabant-treated rat adipocytes, Supplementary Figure S9: Effect of phosphorylation of HSL/LD on Rimonabant-induced lipolysis in the cell-free system, Supplementary Table S1: Effect of cytochalasin B and triacsin C on lipogenesis and lipolysis, Supplementary Table S2: Purification and lipid composition of LD, Supplementary Table S3: PKA-dependent phosphorylation of HSL and LD *in vitro*, Supplementary References 73–83.
