# Supplementary material for: CB1 Receptor-Dependent and Independent Induction of Lipolysis in Primary Rat Adipocytes by the Inverse Agonist Rimonabant (SR141716A)
Source: Molecules. 2020 Feb 18;25(4):896. doi: 10.3390/molecules25040896 (PMC7070561; doi:10.3390/molecules25040896)
Supplement: Supplementary file 1 [file molecules-25-00896-s001.pdf]

Supplementary Figures, Tables and References

CB1 Receptor-Dependent and Independent Induction of Lipolysis in Primary Rat Adipocytes by the Inverse Agonist Rimonabant (SR141716A)

Günter A. Müller, Andreas W. Herling, Susanne Wied and Timo D. Müller

Supplementary Figure S1. Effect of Rimonabant on the protein and lipid composition of LD.
Aim: To assess whether Rimonabant affects the overall composition of LD, rather than the association of LD with HSL and perilipin-1, adipocytes harboring NBD-FA-labelled AG were incubated for a short term with or without Rimonabant, homogenized and fractionated on two-step sucrose gradients. Blotted gradient fractions were probed with antibodies against the typical LD-associated proteins of the PAT family, perilipin-1, adipophilin, TIP47, S3-12 [74-80], the lipid raft and putative LD-associated protein, caveolin-1 [81-83], the endoplasmic reticulum luminal protein, GRP78, the membrane marker, calnexin, and HSL [84].

(**A**) (**B**)

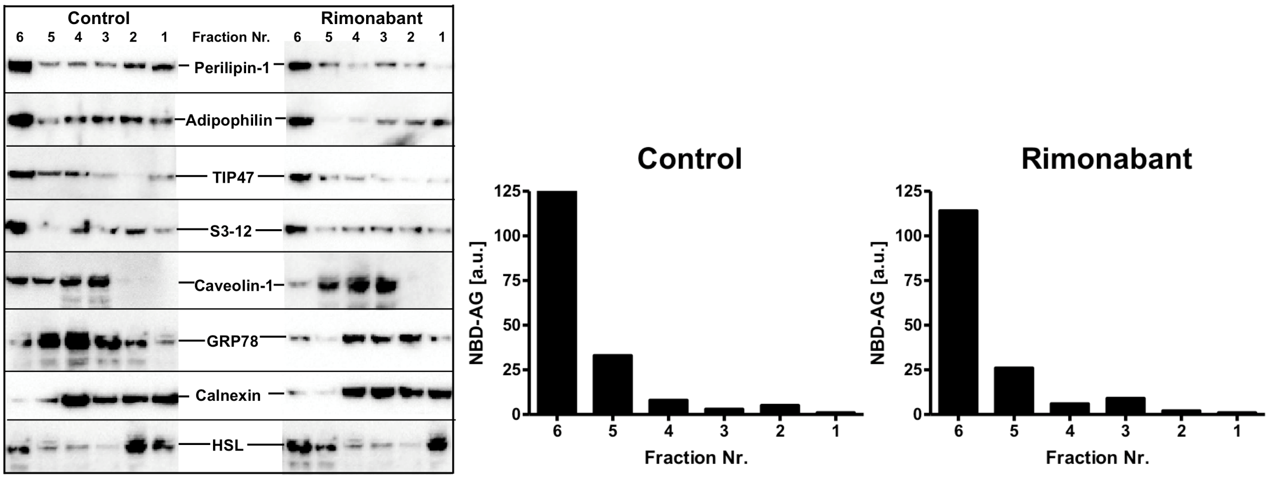

Primary rat adipocytes, which had been metabolically labelled with NBD-FA, were incubated (2 h, 37 °C) in the absence (control) or presence of 10 μM Rimonabant. Subsequently, the homogenate of the washed adipocytes was fractionated by centrifugation through a sucrose cushion (see Materials and Methods). (**A**) Proteins contained in identical volumes of fractions 1-6 were precipitated / extracted and then subjected to SDS-PAGE and immunoblotting, as indicated. The images of a typical experiment are shown repeated once with similar results. (**B**) The amounts of total NBD-FA-labelled AG contained in identical volumes of fractions 1-6 were determined by fluorescence measurement (Spectramax, Molecular Devices).

Results: Analysis of the individual fractions for fluorescence revealed almost exclusive enrichment of NBD-FA-labelled AG with the top fraction 6 apparently harboring the LD (**B**). During the incubation period of 20 min, Rimonabant did not significantly alter the amount of NBD-FA-labelled LD. The major portions of perilipin-1, adipophilin, TIP47 and S3-12 floated at the top of the gradient into fraction 6 in both control and treated cells, with the amount of perilipin-1 decreasing considerably upon Rimonabant treatment, and the distribution of the other LD-associated proteins remaining roughly unaffected. Maximal immunoreactivities of caveolin-1, GRP78 and calnexin were found in the middle gradient fractions 3 and 4. Only for caveolin-1 a considerable portion was recovered with the LD in fraction 6. This is in agreement with recent findings concerning the localization of caveolin-1 at the surface [76-78] or in the core of LD of adipocytes [79] and its implication in LD biogenesis as well as lipolysis [75]. HSL was recovered predominantly with the bottom gradient fractions 2 and 1, together with the soluble cytosolic marker protein lactate dehydrogenase (data not shown). However, significant portions of HSL were also recovered with the LD-containing fraction 6, the amounts of which were elevated upon short-term treatment with Rimonabant (**A**). The data confirm the Rimonabant-induced redistribution of perilipin-1 and HSL (see Figure 2) from and to LD, respectively. Their

expected accompanying reverse accumulation and deprivation, respectively, in the cytosol (fractions 1 and 2) were not observed, presumably due to pronounced dilution along the sucrose gradient.

Conclusion: Rimonabant does not alter the total protein / lipid composition of adipocyte LD in the short-term, as the relative distribution of the other LD-associated proteins tested and their lipid content were not visibly affected. This argues for specific engagement of the lipolytic machinery and its key components, HSL and perilipin-1, by Rimonabant rather than for interference with the biogenesis of LD, which is assumed to be driven by adipophilin, TIP47 and S3-12 as well as caveolin-1 [73,74].

Supplementary Figure S2. Time course of the Rimonabant-induced adipocyte lipolysis.
Aim: The time courses for maximal Rimonabant-induced lipolysis in the absence or presence of a 20-fold molar excess of CP55.490 in NBD-FA-labelled adipocytes were investigated to confirm the operation of two distinct lipolytic responses.

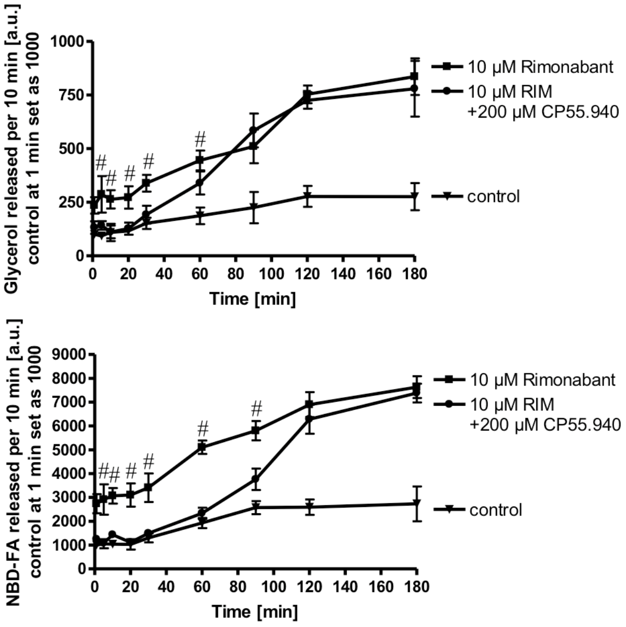

Primary rat adipocytes, which had been metabolically labelled with NBD-FA, were incubated (20 min, 37 °C) in the absence (control ▼, ■) or presence of 200 µM CP55.940 (●) prior to addition of Rimonabant (final concentration 10 µM; ●, ■) or DMSO (control ▼). After further incubation for the periods indicated, 500-µL aliquots of the total incubation mixtures were transferred into 2-mL eppendorf vials and then subjected to centrifugation (500x *g*, 2 min). The infranatant was removed by suction. After addition of 0.5 mL of fresh incubation medium lacking Rimonabant and CP55.940 to the remaining cell layer, the resuspended adipocytes were incubated (10 min, 37 °C). Subsequently, the concentrations of glycerol (upper panel) and NBD-FA (lower panel) released into the incubation medium were assayed enzymatically and by fluorometry, respectively. Mean ± SD of two different cell preparations with incubations in duplicate and measurements in duplicate. # $p \leq 0.01$ for Rimonabant-treated adipocytes between absence and presence of CP55.940

Results: Within 1 min of incubation (*plus* 10 min assay period), Rimonabant induced a 2-fold increase in the releasing rate of both glycerol and NBD-FA, which was completely abrogated by excess of CP55.490. With prolonged incubation with Rimonabant (20 to 180 min), the rate of release gradually increased to 3.5- to 4-fold of the control lipolytic rates. The time-dependent Rimonabant-induced increase in the lipolytic rate was accompanied by a gradual decrease in lipolysis inhibition by CP55.940, which was completely abolished at 120 min.

Supplementary Figure S3. Differential effect of propranolol on the isoproterenol- and Rimonabant-induced adipocyte lipolysis.

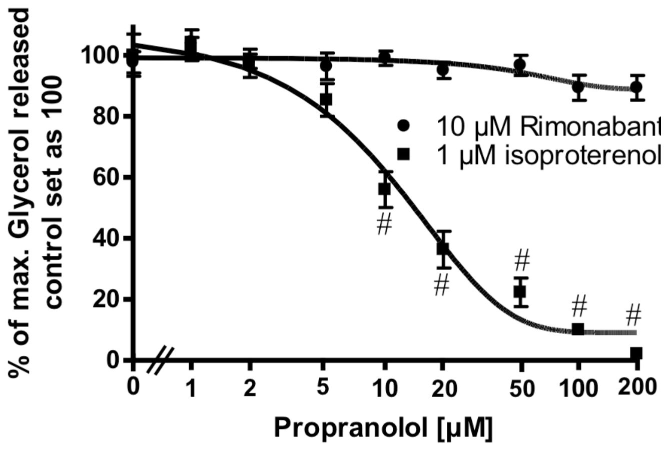

Primary rat adipocytes were incubated (20 min, 37 °C) in the absence or presence of increasing concentrations of propranolol prior to the addition of isoproterenol (final concentration 1 µM; ■) or Rimonabant (final concentration 10 µM; ●). After further incubation (3 h, 37 °C), the concentration of glycerol released into the incubation medium was assayed. Mean ± SD of incubations in triplicate and measurements in duplicate. # $p \leq 0.01$ for propranolol- and then isoproterenol-treated adipocytes *vs.* isoproterenol-treated adipocytes; no significant differences for Rimonabant-treated adipocytes

Supplementary Figure S4. Partial inhibition of Rimonabant-induced adipocyte lipolysis by insulin.

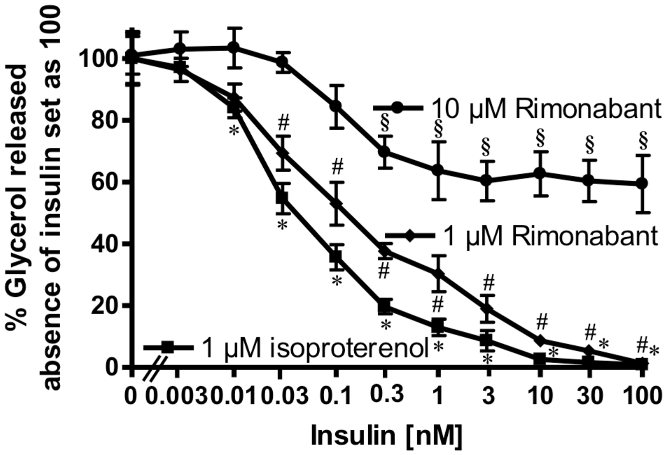

Primary rat adipocytes were incubated (10 min, 37 °C) in the absence or presence of increasing concentrations of human insulin prior to the addition of isoproterenol (final conc. 1 µM; ■) or Rimonabant (final conc. 10 µM; ●, 1 µM; ◆). After further incubation (3 h, 37 °C), the concentration of glycerol released into the incubation medium was assayed. The glycerol concentrations measured in the absence of insulin in response to the lipolytic stimuli were set as 100% in each case. Mean ± SD of two different cell preparations with incubations in triplicate and measurements in duplicate. *, #, § $p \leq 0.01$ for insulin- and then Rimonabant (1 and 10 µM)- / isoproterenol-treated adipocytes *vs.* Rimonabant- / isoproterenol-treated adipocytes

Supplementary Figure S5. Stimulation of lipolysis in adipocytes from Rimonabant-treated rats.

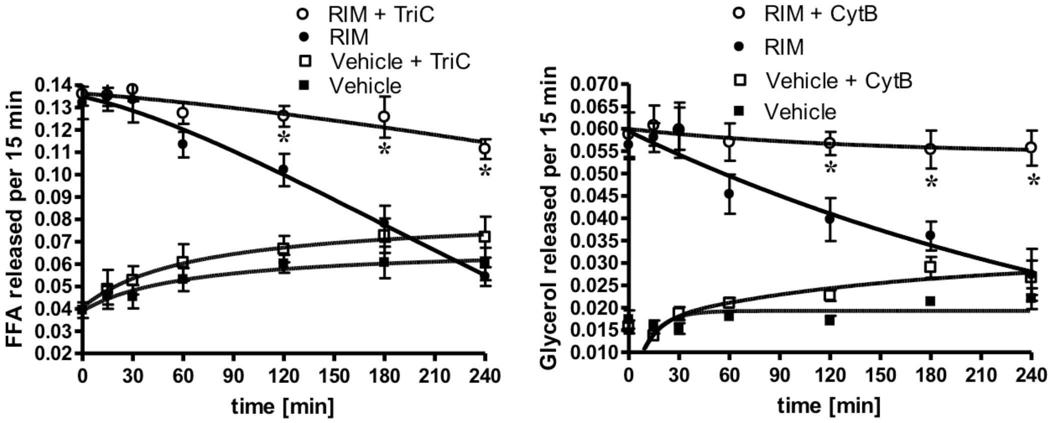

Male Wistar rats (280-320 g) were treated with one dose of Rimonabant (30 mg / kg, *p.o.*; RIM) or vehicle. After 5 h under free access to food epididymal adipocytes were prepared by collagenase digestion and washed. Primary adipocytes isolated from Rimonabant- or vehicle-treated rats were incubated (37 °C) in medium harboring 10 mM glucose and 0.1 nM insulin in the absence or presence of cytochalasin B (10 μM; CytB) or triacsin C (30 μM; TriC) for increasing periods of time. Subsequently, the adipocytes were washed by flotation, then resuspended in fresh medium and incubated (15 min, 37 °C). Thereafter, the amounts of glycerol and FFA released into the incubation medium were assayed enzymatically. Mean $\pm$ SD of two different adipocyte / LD preparations with incubations / measurement in quadruplicate. * $p \leq 0.01$ for adipocytes from Rimonabant-treated rats between absence and presence of TriC or CytB; no significant differences for vehicle-treated rats

Supplementary Figure S6. Stimulation of lipolysis in a cell-free system by Rimonabant.

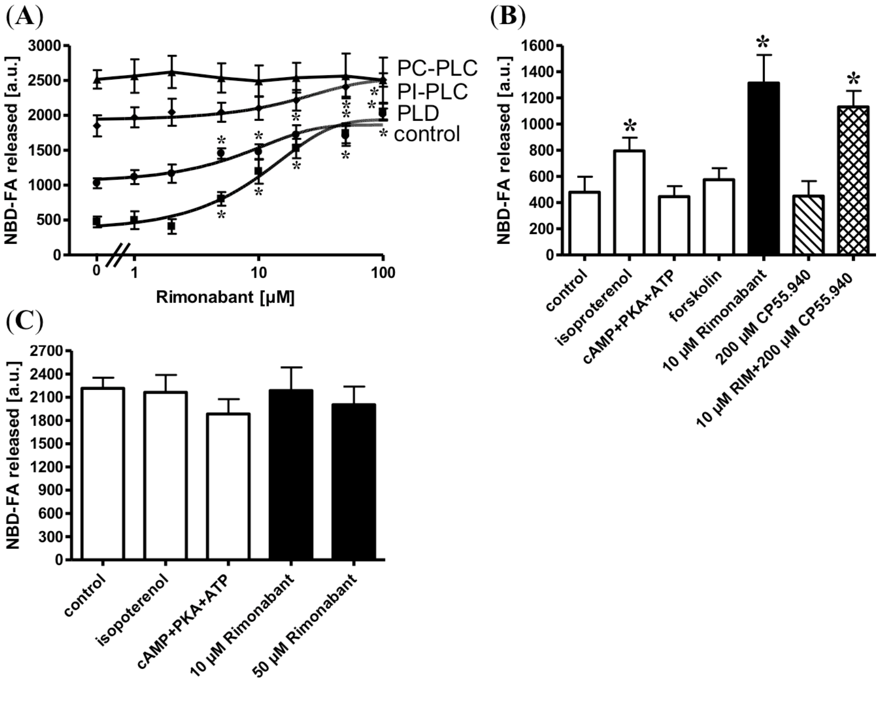

LD prepared from untreated washed rat adipocytes, which had been metabolically labelled with NBD-FA, were reconstituted with partially purified rat adipocyte HSL (see Materials and Methods) and then incubated (30 °C, 90 min) without (**A**,**B**) or with gum arabic and ultrasonic treatment (5 min, ultrasonic bath incubator, full power; **C**), in the absence (control) or presence of 1 μM isoproterenol or 50 μM cAMP, 150 ng PKA and 500 μM ATP, 100 nM forskolin, or Rimonabant alone or in combination with CP55.940, as indicated (**B**,**C**), or in the absence or presence of increasing concentrations of Rimonabant without (control) or with PLD (cabbage, 3 U / mL) or PI-PLC (*Bacillus cereus*, 0.5 U / mL) or PC-PLC (*Bacillus thuringiensis*, 1 U / mL; **A**). The organic phase of the chloroform / methanol / HCl-extract of the total incubation mixture was analyzed by TLC. The amount of NBD-FA lipolytically released from the NBD-FA-labelled AG1-

4 and run in parallel to a synthetic NBD-FA marker was quantitatively evaluated by fluorescence imaging. Mean ± SD of two different LD preparations with incubations in triplicate. * $p \leq 0.05$ for phospholipase-treated / control and then Rimonabant-treated LD *vs.* phospholipase-treated / control LD (**A**), no significant differences for PC-PLC; * $p < 0.01$ for treated LD (as indicated) *vs.* control LD (**B**); no significant differences for LD treated with gum arabic and ultrasonication (**C**)

Supplementary Figure S7. Effect of Rimonabant on HSL activity *in vitro*.

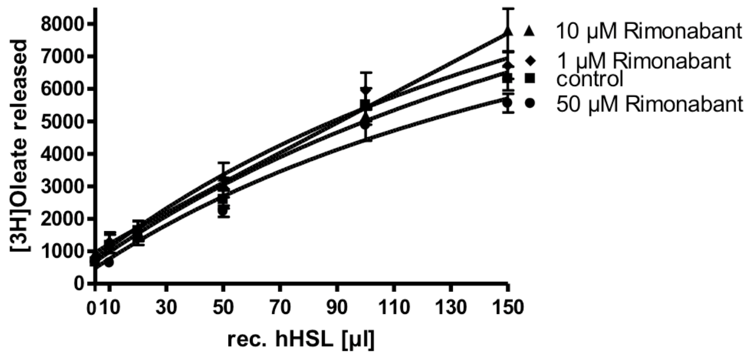

Increasing amounts of recombinant human HSL were incubated (2 h, 30 °C) with emulsified [^{3}H]trioleoylglycerol in the absence (control) or presence of Rimonabant at the concentrations indicated. After adjustment to alkaline pH, the incubation mixture was partitioned between an organic and aqueous phase. The [^{3}H]oleate released was determined in the aqueous phase by liquid scintillation counting. Mean ± SD of three different incubations with measurements in duplicate. No significant differences between absence and presence of Rimonabant

Supplementary Figure S8. Stimulation of lipolysis in a cell-free system from Rimonabant-treated rat adipocytes.

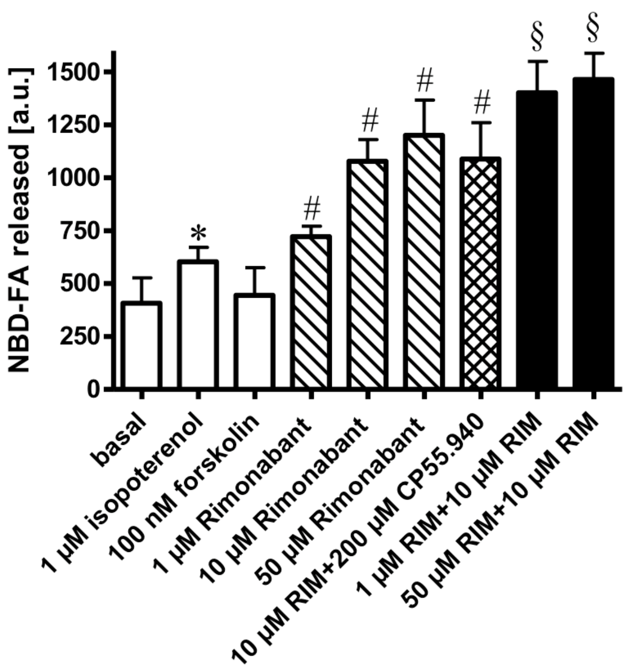

Primary rat adipocytes, which had been metabolically labelled with NBD-FA, were incubated (20 min, 37 °C) in the absence (basal) or presence of isoproterenol (1 µM), forskolin (100 nM)(open bars), Rimonabant alone (hatched bars) or in combination with CP55.940 (200 µM, double-hatched bars). Subsequently, LD were prepared from the washed adipocytes, reconstituted with partially purified rat adipocyte HSL (copurified+exogenous HSL)(see Materials and Methods) and then incubated (90 min, 30 °C) in the absence (open, hatched, double-hatched bars) or presence (filled bars) of 10 µM Rimonabant. The organic phase of the chloroform / methanol / HCl-extract of the total incubation

mixtures was analyzed by TLC. The amount of NBD-FA lipolytically released from the NBD-FA-labelled AG1-4 and run in parallel to a synthetic NBD-FA marker (ma) was quantitatively evaluated by fluorescence imaging. Mean ± SD of two different LD preparations with incubations in duplicate. Values from reactions lacking exogenously added HSL were subtracted in each case. *, #, § $p \leq 0.05$ for Rimonabant-treated LD from treated adipocytes (as indicated) *vs*. untreated LD from basal adipocytes

Supplementary Figure S9. Effect of CP55.490 on Rimonabant stimulation of lipolysis in a cell-free system

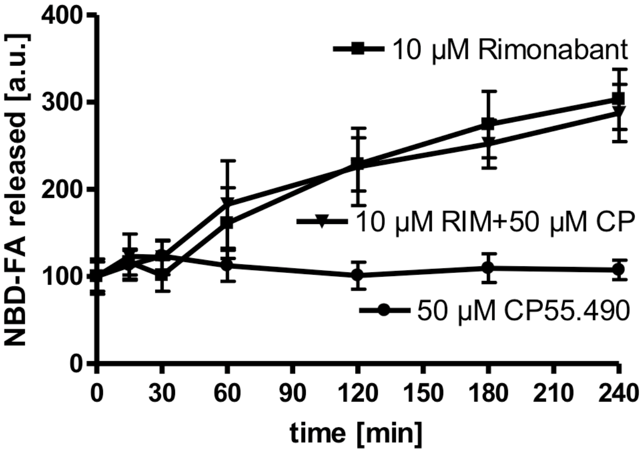

Primary rat adipocytes, which had been metabolically labelled with NBD-FA, were incubated (37 °C) with 10 µM Rimonabant (■), 50 µM CP55.490 (●) or 10 µM Rimonabant *plus* 50 µM CP55.490 (▼) for increasing periods of time. Subsequently, LD were prepared from the washed adipocytes, reconstituted with recombinant human HSL (see Materials and Methods) and then incubated (90 min, 30 °C). After extraction of the total incubation mixtures with chloroform / methanol / HCl, the organic phases were analyzed by TLC as described (Figure 11). The amount of NBD-FA lipolytically released from the NBD-FA-labelled AG1-4 was quantitatively evaluated by fluorescence imaging (mean ± SD of incubations in quadruplicate and TLC analysis in duplicate. NBD-FA released in the "HSL + LD" (absence of Rimonabant during cell-free lipolysis and adipocyte incubation, respectively) was set as 100. Values from corresponding reactions lacking exogenously added HSL were subtracted in each case in order to correct for HSL copurifying with the LD. Thus, the lipolysis activity depicted as NBD-FA released relies on the exogenously added recombinant human HSL rather than endogenous LD-associated rat HSL. No significant differences for LD from Rimonabant-treated adipocytes between absence and presence of CP55.490

Supplementary Tables

Supplementary Table S1. Effect of cytochalasin B and triacsin C on lipogenesis and lipolysis.

		**Lipid Synthesis**		**Lipolysis**	
	**Time [h]**	**low glucose**	**low insulin high glucose**	**low glucose**	**low glucose isoproterenol**
**Control**	1	3754 ± 273	79433 ± 3140	132 ± 16	1165 ± 204
	4	12841 ± 897	295232 ± 9035	497 ± 50	4238 ± 513
**CytB**	1	944 ± 112*	8126 ± 376*<br>**89.8 %**	102 ± 20	994 ± 152<br>**14.7 %**
	4	1341 ± 190*	11427 ± 298*<br>**96.1 %**	413 ± 53	3562 ± 422<br>**16.0 %**
**TriC**	1	1783 ± 199*	13207 ± 672*<br>**85.4 %**	162±35	1013 ± 199<br>**13.1 %**
	4	2659 ± 324*	18942 ± 811*<br>**93.6 %**	541 ± 66	3894 ± 231<br>**8.1 %**

Primary rat adipocytes were incubated (10 min, 37 °C) in the absence (Control) or presence of cytochalasin B (10 μM; CytB) or triacsin C (30 μM; TriC). Portions of the cells were incubated (37 °C) for 1 or 4 h with low (0.1 mM) glucose or with low (0.1 nM) insulin and high (10 mM) glucose or with 1 μM isoproterenol and 0.1 mM glucose and then assayed for lipid synthesis as incorporation of [^{3}H]glucose into total cellular acylglycerols or lipolysis as release of glycerol into the incubation medium (see Materials and Methods). Mean ± SD from 3 independent adipocyte preparations with measurements in duplicate for lipid synthesis (dpm, corrected for identical specific [^{3}H]glucose radioactivity) and triplicate for lipolysis (a. u.). % inhibition of lipid synthesis at high glucose/low insulin or isoproterenol-stimulated lipolysis by Cyt B or TriC in bold. * $p \leq 0.01$ *vs.* control for the same incubation time

Supplementary Table S2. Purification and lipid composition of LD.

	**Control**			**RIM 20 min**	**RIM 180 min**
**Lipid Class**	**Homogenate**	**LD**	**LD / Hom**	**LD**	**LD**
FFA	20.9 ± 3.1	6.5 ± 0.9	0.31	5.9 ± 0.5	5.1 ± 1.0
Cholesterol	96.2 ± 12.2	2.9 ± 0.6	0.03	3.5 ± 0.7	3.9 ± 0.5
Phospholipid	207 ± 29.5	0.5 ± 0.2	0.002	0.4 ± 0.2	0.2 ± 0.1
DAG	1.3 ± 0.5	2.1 ± 0.4	1.61	1.4 ± 0.3	1.8 ± 0.6
TAG	25.5 ± 2.6	39.6 ± 2.5	1.56	45.8 ± 6.2	37.8 ± 5.0
Cholesteryl Ester	3.5 ± 0.5	5.8 ± 0.8	1.66	4.5 ± 0.7	4.9 ± 0.4
SL/CL	10.72	0.21	-	0.19	0.21

Total homogenates and LD were prepared from primary rat adipocytes which had been incubated (20 or 180 min) in the absence (control) or presence of 10 μM Rimonabant (RIM). Lipid and protein extracts were analyzed for the content of FA, phospholipid, cholesterol, cholesterylester, DAG, TAG and protein, respectively (see Materials and Methods). Values represent means (nmol / mg protein) ± SD of three independent preparations and measurements in triplicate. The

ratios of the lipid contents between LD and homogenate (Hom) and between LD surface lipid (SL = FA + phospholipid + cholesterol) and LD core lipid (CL = DAG + TAG + cholesteryl ester) are given.

Results: Whereas no lipid species was detected unique to LD, as compared with adipocyte homogenate, LD were relatively deficient in SL (LD / Hom < 1 for each lipid class) and rich in CL (LD / Hom > 1), resulting in a SL/CL ratio of 0.2 and 10.7 for LD and the homogenate, respectively. These low levels of SL recovered with LD (17% of the total lipids) indicate their considerable purity since membranes of other organelles harbour much higher levels of SL. Contamination from other membranes would have led to increased levels of total SL and the SL/CL ratio measured for the isolated LD. Among the CL (83% of the total lipids), the main constituent TAG was 6.5-fold more prominent than cholesteryl ester (see Supplementary Table 2). Remarkably, among the SL, FA were most prominent (66%). This high portion of FA compared to cholesterol and phospholipids in the SL of LD may be due to lipolysis ongoing during the isolation and purification of the LD. In fact, a small but significant amount of HSL was found to remain associated with isolated LD (see below). Digestion of the CL of LD by residual HSL or other lipases would also explain the considerable amount of DAG found within the CL.

Conclusion: The lipid composition of the LD prepared from the primary rat adipocytes is typical for the large TAG-rich LD from adipose tissue [36]. The nature and integrity of the LD were further confirmed by the demonstration of co-fractionation of the major LD-associated proteins identified for adipocytes so far. Rimonabant had no significant effect on the lipid composition of LD upon incubation of intact adipocytes for short and long term (Supplementary Table 2) or of isolated LD (data not shown) as reflected in the unaltered total distribution of the lipid constituents and constant SL/CL ratios of the LD. This is in agreement with the unaltered amount of NBD-FA-labelled AG recovered with LD after lipolysis stimulation by Rimonabant in intact rat adipocytes (see Figure 3B).

Supplementary Table S3. PKA-dependent phosphorylation of HSL and LD *in vitro*.

	control	PKA	PKA + PKI	PKA + RIM
**HSL**	104 ± 37	2375 ± 405*	285 ± 55#	1995 ± 351
**LD**	162 ± 42	982 ± 188*	266 ± 70#	845 ± 101
**RIM-LD**	133 ± 50	1142 ± 295*	210 ± 59#	911 ± 169
**HSL+LD**	198 ± 29	1971 ± 348*	192 ± 108#	1688 ± 250

Recombinant human HSL, LD prepared from untreated (LD) or Rimonabant (10 μM, 3 h, 37 °C)-treated (RIM-LD) rat adipocytes or HSL *plus* LD were incubated (HSL, 4 °C; LD, 30 °C; HSL + LD, 4 °C) with 0.1 mM [γ-^{32}P]ATP in the presence or absence (control) of PKA with or without a 4-fold molar excess of protein kinase A inhibitor peptide (PKI) or 10 μM Rimonabant (RIM)(see Materials and Methods). After addition of EDTA (final conc. 5 mM), proteins of total incubation mixtures were precipitated with TCA / acetone. Collected precipitates were washed, dissolved and counted for radioactivity. Mean [dpm] ± SD of three incubations with measurements in duplicate. * $p \leq 0.01$ for PKA *vs.* control, # $p \leq 0.01$ for PKA in the presence *vs.* absence of PKI, no significant differences for PKA in the presence *vs.* absence of Rimonabant

Supplementary References

74. Brasaemle, D.L.; Barber, D.L.; Wolins, N.E.; Serrero, G.; Blanchette-Mackie, E.J.; Londos, C. Adipose differentiation-related protein is an ubiquitously expressed lipid storage droplet-associated protein. *J. Lipid. Res.* **1997**, *38*, 2249-2263.

75. Robenek, H.; Robenek, M.J.; Buers, I.; Lorkowski, S.; Hofnagel, O.; Troyer, D.; Severs, N.J. Lipid droplets gain PAT family proteins by interaction with specialized plasma membrane domains. *J. Biol. Chem.* **2005**, *280*, 26330-26338.

76. Robenek, H.; Robenek, M.J.; Troyer, D. PAT family proteins pervade lipid droplet cores. *J. Lipid Res.* **2005**, *46*, 1331-1338.

77. Cohen, A.W.; Razani, B.; Schubert, W.; Williams, T.M.; Wang, X.B.; Lyengar, P.; Brasaemle, D.L.; Scherer, P.E.; Lisanti, M.P. Role of caveolin-1 in the modulation of lipolysis and lipid droplet formation. *Diabetes* **2004**, *53*, 1261-1270.

78. Nishimoto, Y.; Tamori, Y. CIDE Family-mediated unique lipid droplet morphology in white adipose tissue and brown adipose tissue determines the adipocyte energy metabolism. *J. Atheroscler. Thromb.* **2017**, *24*, 989-998.

79. Fujimoto, T.; Kogo, H.; Ishiguro, K.; Tauchi, K.; Nomura, R. Caveolin-2 is targeted to lipid droplets, a new "membrane domain" in the cell. *J. Cell Biol.* **2001**, *152*, 1079-1086.

80. Cohen, S. Lipid droplets as organelles. *Int. Rev. Cell. Mol. Biol.* **2018**, *337*, 83-110.

81. Sztalryd, C.; Xu, G.D.; Contreras, J.A.; Kimmel, A.R.; Londos, C. Perilipin A is essential for the translocation of hormone-sensitive lipase during lipolytic activation of adipocytes. *J. Cell Biol.* **2002**, *161*, 1103-1113.

82. Pol, A.; Luetterforst, R.; Lindsay, M.; Heino, S.; Ikonen, E.; Parton, R.G. A caveolin dominant negative mutant associates with lipid bodies and induces intracellular cholesterol imbalance. *J. Cell Biol.* **2001**, *152*, 1057-1070.

83. Ostermeyer, A.G.; Paci, J.M.; Zeng, Y.; Lublin, D.M.; Munro, S.; Brown, D.A. Accumulation of caveolin in the endoplasmic reticulum redirects the protein to lipid storage droplets. *J. Cell Biol.* **2002**, *152*, 1071-1078.

84. Robenek, M.J.; Severs, N.J.; Schlattmann, K.; Plenz, G., Zimmer, K.-P.; Troyer, D.; Robenek, H. Lipids partition caveolin-1 from ER membranes into lipid droplets: updating the model of lipid droplet biogenesis. *FASEB J.* **2004**, *18*, 866-868.
